# A holo-spectral EEG analysis provides an early detection of cognitive decline and predicts the progression to Alzheimer’s disease

**DOI:** 10.3389/fnagi.2023.1195424

**Published:** 2023-08-22

**Authors:** Kwo-Ta Chu, Weng-Chi Lei, Ming-Hsiu Wu, Jong-Ling Fuh, Shuu-Jiun Wang, Isobel T. French, Wen-Sheng Chang, Chi-Fu Chang, Norden E. Huang, Wei-Kuang Liang, Chi-Hung Juan

**Affiliations:** ^1^Institute of Cognitive Neuroscience, National Central University, Taoyuan, Taiwan; ^2^Yang-Ming Hospital, Taoyuan, Taiwan; ^3^Cognitive Intelligence and Precision Healthcare Center, National Central University, Taoyuan, Taiwan; ^4^Division of Neurology, Department of Internal Medicine, Chi Mei Medical Center, Tainan, Taiwan; ^5^Department of Long-Term Care and Health Promotion, Min-Hwei Junior College of Health Care Management, Tainan, Taiwan; ^6^Department of Neurology, Neurological Institute, Taipei Veterans General Hospital, Taipei, Taiwan; ^7^School of Medicine, College of Medicine, National Yang Ming Chiao Tung University, Taipei, Taiwan; ^8^Taiwan International Graduate Program in Interdisciplinary Neuroscience, National Central University and Academia Sinica, Taipei, Taiwan; ^9^Key Laboratory of Data Analysis and Applications, First Institute of Oceanography, SOA, Qingdao, China; ^10^Department of Psychology, Kaohsiung Medical University, Kaohsiung, Taiwan

**Keywords:** resting state EEG (rsEEG), Holo-Hilbert spectral analysis (HHSA), machine learning, mild cognitive impairment (MCI), amplitude modulation (AM)

## Abstract

**Aims:**

Our aim was to differentiate patients with mild cognitive impairment (MCI) and Alzheimer’s disease (AD) from cognitively normal (CN) individuals and predict the progression from MCI to AD within a 3-year longitudinal follow-up. A newly developed Holo-Hilbert Spectral Analysis (HHSA) was applied to resting state EEG (rsEEG), and features were extracted and subjected to machine learning algorithms.

**Methods:**

A total of 205 participants were recruited from three hospitals, with CN (*n* = 51, MMSE > 26), MCI (*n* = 42, CDR = 0.5, MMSE ≥ 25), AD1 (*n* = 61, CDR = 1, MMSE < 25), AD2 (*n* = 35, CDR = 2, MMSE < 16), and AD3 (*n* = 16, CDR = 3, MMSE < 16). rsEEG was also acquired from all subjects. Seventy-two MCI patients (CDR = 0.5) were longitudinally followed up with two rsEEG recordings within 3 years and further subdivided into an MCI-stable group (MCI-S, *n* = 36) and an MCI-converted group (MCI-C, *n* = 36). The HHSA was then applied to the rsEEG data, and features were extracted and subjected to machine-learning algorithms.

**Results:**

(a) At the group level analysis, the HHSA contrast of MCI and different stages of AD showed augmented amplitude modulation (AM) power of lower-frequency oscillations (LFO; delta and theta bands) with attenuated AM power of higher-frequency oscillations (HFO; beta and gamma bands) compared with cognitively normal elderly controls. The alpha frequency oscillation showed augmented AM power across MCI to AD1 with a reverse trend at AD2. (b) At the individual level of cross-sectional analysis, implementation of machine learning algorithms discriminated between groups with good sensitivity (Sen) and specificity (Spec) as follows: CN elderly vs. MCI: 0.82 (Sen)/0.80 (Spec), CN vs. AD1: 0.94 (Sen)/0.80 (Spec), CN vs. AD2: 0.93 (Sen)/0.90 (Spec), and CN vs. AD3: 0.75 (Sen)/1.00 (Spec). (c) In the longitudinal MCI follow-up, the initial contrasted HHSA between MCI-S and MCI-C groups showed significantly attenuated AM power of alpha and beta band oscillations. (d) At the individual level analysis of longitudinal MCI groups, deploying machine learning algorithms with the best seven features resulted in a sensitivity of 0.9 by the support vector machine (SVM) classifier, with a specificity of 0.8 yielded by the decision tree classifier.

**Conclusion:**

Integrating HHSA into EEG signals and machine learning algorithms can differentiate between CN and MCI as well as also predict AD progression at the MCI stage.

## Graphical Abstract

A reference guide for early detection of MCI and AD. 
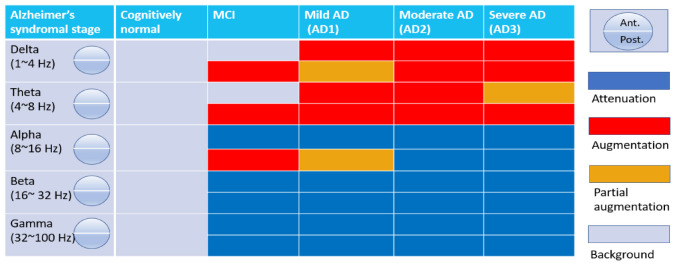

## 1. Introduction

### 1.1. Alzheimer’s disease and the DIAN study

Alzheimer’s disease (AD) is a chronic, debilitating neurodegenerative disorder that manifests progressive cognitive decline with behavioral and psychological symptoms until loss of independence in daily activities, accounting for the leading cause of dementia. The Dominantly Inherited Alzheimer’s Network (DIAN) study demonstrates the natural history of familial AD (Bateman et al., 2012), constructed by cross-sectional biomarker data collection, including global cognitive score, fluid biomarkers, neuroimaging, and structural biomarkers coupled with the corresponding estimation of years from expected symptom onset. Depending on the natural history, amyloid-beta deposition may exist 25 years before symptom onset, ensuing in progressive synaptic dysfunction and loss in vulnerable brain areas (Fu et al., 2018). These pathologies can be measured by altered glucose metabolism (FDG-PET; Herholz, 2010) and altered brain oscillations (EEG or MEG; Cook and Leuchter, 1996). However, the exact characteristics of neurophysiological signatures (e.g., EEG) in the AD progression timeframe need further investigation as these features may represent synaptic dysfunction in the early stage or severe neuronal loss in later disease stages (Smailovic and Jelic, 2019; Petrova et al., 2020). Therefore, EEG biomarkers during prodromal AD can be a potential target for preventive interventions or a monitoring tool for pharmacological therapy (Babiloni et al., 2006a,^2013^; McDade and Bateman, 2017).

The National Institute on Aging and Alzheimer’s Association (NIA-AA) proposed a flexible AT(N) research framework for AD in 2018, which defines the neurobiological profile of Alzheimer’s disease continuum and welcomes new biomarkers to be added to the system upon clinical validation (Jack et al., 2018). Based on this framework, we use a cross-sectional rsEEG dataset following each AD category to construct stage-wise characteristic-altered brain oscillations. The clinical staging in the AD continuum grades from one to six, where stages 3, 4, 5, and 6 correspond to mild cognitive impairment (MCI), mild AD (AD1), moderate AD (AD2), and severe AD (AD3; Jack et al., 2018), respectively.

### 1.2. Resting-state EEG as a neurophysiological biomarker for the diagnosis of AD

Resting-state electroencephalographic (rsEEG) signals provide biomarkers for the early diagnosis of MCI and AD, where four major features are observed, including slowing and reduced complexity of EEG signals, aberrant synchronization measures (Dauwels et al., 2010), and neuro-modulatory deficits in amplitude modulation (AM) of EEG oscillations (Falk et al., 2012). To depict the time-frequency diagram of EEG signals, the Hilbert-Huang transform (HHT) can be applied to efficiently handle non-stationary EEG signals based on the Empirical Mode Decomposition (EMD; Huang et al., 1998). The Holo-Hilbert Spectral Analysis (HHSA) is an extension of the HHT that provides AM information on each carrier frequency (*f*c) band in addition to the time-frequency (Huang et al., 2016). Conventional spectral-based EEG analyses such as the windowed Fourier, complex wavelet, and Hilbert transform require *a priori* knowledge to define the frequency bin of different frequency bands before band-pass filtering. However, the HHSA uses an adaptive model-free EMD approach to decipher raw EEG signals into intrinsic mode functions (IMFs) before defining the frequency bin of corresponding frequency bands upon a dyadic rule, namely, the dyadic filter-bank property of IMF (Flandrin et al., 2004). In this study, we used dyadic frequency bins to demonstrate the results since they were close to the natural bandwidths of IMFs. Meaningful frequency bands are more appropriately defined as *a posteriori*. Other issues in the pre-specified narrowband have been scrutinized using the FOOOF algorithm (Donoghue et al., 2020), demonstrating the implications of parameterizing neural power spectra into aperiodic and periodic components on cognition and physiological conditions. We used the FOOOF algorithm to examine our data, which showed consistent results (Figure 3). Compared with FOOOF, HHSA does not assume a model behind the signal. The categorization and interpretation of the data spectral representations rely on subsequent statistical analyses. The main advantage of using HHSA over conventional signal processing is its ability to characterize non-linear interactions across different oscillatory components (Huang et al., 2016; Nguyen et al., 2019; Juan et al., 2021; Liang et al., 2021).

### 1.3. Unmet needs of current rsEEG analysis in the classification between MCI and AD and in the prediction from MCI to AD

Previous EEG reports only provide partial results concerning cognitive decline and AD, chiefly emphasizing an increment in LFO power density and a decrement in HFO power density in AD (van Straaten et al., 2014). For example, reports of altered oscillations in studies using LORETA-based source activity used predefined frequency bands to discriminate between neurodegenerative diseases, with the highest frequency band limited to beta2 (20∼30 Hz; Babiloni et al., 2006b,^2007^, ^2011^). Two other studies were also restricted to beta (12∼19.5 Hz) and beta3 (24∼29.99 Hz) (Musaeus et al., 2018a; Smailovic et al., 2018), where higher gamma-band signals were omitted because of intangible high-frequency noise derived from the environment and muscle artifacts (Whitham et al., 2008; Widmann and Schröger, 2012). Although previous EEG studies compare MCI and AD with CN (Jelic et al., 1996; Babiloni et al., 2006b,^2017^, ^2018^; Musaeus et al., 2018a; Nakamura et al., 2018), there is a lack of cross-sectional survey of EEG signatures across MCI to each AD subgroup. By utilizing the HHSA, a complete display of the power relationship between AM frequency (*f*am) and carrier frequency (*f*c) with a topographical description of the power density, specifically a full dimensional frequency spectrum of EEG signals (i.e., *f*c, *f*am, and time; Huang et al., 2016), is acquired. Extracted features can then be deployed in machine-learning algorithms to classify disease and predict disease progression (Falk et al., 2012; Fraga et al., 2013), as the additional dimension of features empowers the algorithm’s performance (i.e., *f*am in the HHSA) in the training stage (Falk et al., 2012).

### 1.4. Study aims

Firstly, cross-sectional group-level HHSA rsEEG in the MCI and each AD subgroup were compared with CN to construct the EEG signal changes from the MCI to AD subgroups, where the EEG signatures of the MCI and AD subgroups were hypothesized to display stage-wise progressive changes compared to CN. Secondly, we differentiated patients in the MCI and AD subgroups from the CN group by extracting features from the conventional methods and HHSA for further deployment in machine learning algorithms. Thirdly, we prospectively followed up on the MCI group within 3 years, resulting in the MCI-converted (MCI-C) and MCI-stable (MCI-S) subgroups. Using inter-group comparisons of baseline HHSA EEG, group-levels were delineated using statistically significant differences utilizing the abovementioned feature extraction approach, combined with machine-learning algorithms to predict which subgroup of patients would progress to AD.

## 2. Materials and methods

In this cross-sectional retrospective study, 205 participants were recruited from three hospitals in Taiwan; Yang-Ming Hospital in Taoyuan city, Chi-Mei Medical Centre in Tainan city, and Taipei Veterans General Hospital. All participants’ rsEEGs were recorded in the awake eyes-closed (EC) condition for 6 min. Clinically, probable AD diagnosis was made following the National Institute of Neurological and Communicative Disorders and Stroke–Alzheimer’s Disease and Related Disorders Association (McKhann et al., 1984) and DSM-IV-TR criteria (Segal, 2010). Disease progression severity was categorized using the Chinese version of the Mini-Mental State Examination (MMSE; Folstein et al., 1975) and Clinical Dementia Rating (CDR) score (Lin and Liu, 2003). Participants were subdivided into five groups based on MMSE and CDR score, namely, CN (*n* = 51, MMSE > 26), MCI (*n* = 42, CDR = 0.5, MMSE ≥ 25), AD1 (*n* = 61, CDR = 1, MMSE < 25), AD2 (*n* = 35, CDR = 2, MMSE < 16), and AD3 (*n* = 16, CDR = 3, MMSE < 16) The inclusion criteria in the MCI longitudinal cohort study was a CDR 0.5 score with at least two rsEEG recordings within a 3-year follow-up, yielding 72 participants. The demographics of all participants are shown in Table 1 (See Supplementary Figure 2 for grouping rationale). The average age (i.e., years) of the CN was 69.6 ± 5.8, whereas the average age across the AD subgroups was MCI, 73.5 ± 8.2; AD1, 78.9 ± 6.5; AD2, 80.9 ± 5.2; AD3, 80.1 ± 15.9, respectively. The MMSE score revealed a statistically significant difference among the CN and AD subgroups. The ratio of dataset partitioning for training and testing was approximately 1.47∼3.

**TABLE 1:** Demographic features of cognitively normal subjects (CN), MCI, and AD subgroups.

	CN (*n* = 51)	MCI (*n* = 42)	AD1 (*n* = 61)	AD2 (*n* = 35)	AD3 (*n* = 16)
Gender (Male/Female)	27/24	21/21	30/31	13/22	3/13
Age in years, mean (std)	69.6 (5.8)	73.5 (8.2)	78.9 (6.5)	80.9 (5.2)	80.1 (5.9)
MMSE, mean (std)	28.5 (2.7)*	26.5 (1.4)*	18.2 (3.1)*	11.9 (2.3)*	5.4 (1.8)*
CDR	NA	0.5	1	2	3
Dataset partition (Training/Testing)	31/20	25/17	44/17	21/14	12/4

CN, cognitively normal subjects; MCI, mild cognitive impairment; AD1, mild AD; AD2, moderate AD; AD3, severe AD; CDR, Clinical Dementia Rating; MMSE, Mini-Mental State Examination; NA, not available. *Asterisk denotes statistically significant differences based on one-way ANOVA and Tukey HSD *post hoc* pair-wise comparisons.

### 2.1. Workflow of EEG signals analysis

**FIGURE 1:**
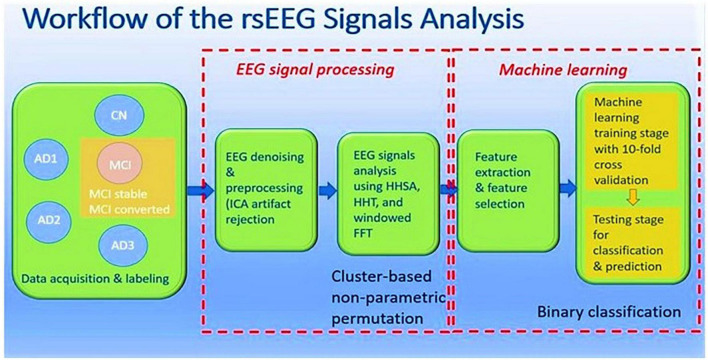
Workflow of rsEEG signals analysis.

The rsEEG data was acquired from both CN and patients, where MCI and AD patients were further categorized into four subgroups: MCI, AD1, AD2, and AD3 (Jack et al., 2018). EEG denoising and preprocessing were performed and subjected to windowed FFT, HHT, and HHSA to decompose and display their spectral and spectro-temporal features, respectively. At individual-level analyses, linear and non-linear features were extracted from these methods and deployed in machine learning algorithms to differentiate between patients with MCI and AD subgroups from CN individuals. Subsequently, the rsEEG analysis was conducted in the pre-specified MCI group (CDR = 0.5) and was followed up on prospectively within 3 years. Two subgroups were categorized according to the clinical labeling: MCI-S and MCI-C. Group-level comparisons were then carried out between and within groups. The same approach was applied to the individual-level analysis by deploying machine-learning algorithms to discriminate rsEEG signals between groups at baseline, the basis of which was to predict which group would progress to AD. The workflow is depicted in Figure 1.

### 2.2. EEG acquisition protocol and signals processing

EEG data acquisition was performed using the Nihon Kohden amplifier with 19 electrode placements according to the international 10–20 system (Supplementary Figure 1). Electrodes were saturated with Ag/AgCl gel and placed on all participants’ heads. The whole 6 min for both eye-closed (EC) and eye-opened (EO) rsEEG was digitized at a 200 Hz sampling rate with an online notch filter at 60 Hz. The reference was the average of electrodes at the two sides of the linked-ear referential electrodes (A1 and A2). Two pairs of bipolar electrodes were also mounted to detect eye movements with the VEOU and VEOL electrodes placed above and below the left eye, respectively, with the HEOR and HEOL electrodes positioned adjacent to the canthus of each eye. Impedances of all channels were maintained below 5 kΩ.

#### 2.2.1. EEG recording, preprocessing, and denoising

The recorded EEG data were fragmented into consecutive epochs of 7,000 ms. Epochs with ocular, muscular, and other types of artifacts were preliminarily identified and excluded by a computerized procedure using Independent Component Analysis (ICA; Gilberet et al., 2017). Finally, the windowed FFT, HHT, and HHSA were applied to compute the power spectrum of each trial. The frequency bins in FFT were defined as delta (0.5∼4 Hz), theta (4.5∼8 Hz), alpha (8.5∼12 Hz), beta (12.5∼30 Hz), and gamma (30.5∼ 80 Hz, Fraga et al., 2013).

#### 2.2.2. HHSA for EEG recordings–The concept of carrier frequency and AM in HHSA

The HHSA (Huang et al., 2016; Nguyen et al., 2019), developed upon the EMD (Huang et al., 1998), was applied to the pre-processed EEG signals. The EMD is an adaptive time-frequency decomposition method that utilizes the local information of the signal to decompose the data into a set of intrinsic mode functions (IMF), defined as a function with the following properties: (1) the number of local extrema (including local maxima and minima) and the number of zero-crossings that are either equal to or differ at most by one; and (2) the mean value of the envelope estimated by local maxima and local minima to be zero (Huang et al., 1998). Essentially, EMD serves as a dyadic filter bank to the data, i.e., each IMF represents signal information in different log2 time scales (Flandrin et al., 2004). The instantaneous frequency and amplitude of each IMF are calculated from a direct quadrature transform (Huang et al., 2009), which constitutes the HHT and generates a high-resolution time-carrier frequency spectral representation, where the carrier frequency is comparable to conventional narrowband frequency. The envelope (i.e., amplitude function) of each given IMF is acquired by fitting along the maxima of the absolute-valued IMF using a cubic spline algorithm (Huang et al., 2009). Through the application of the second-layer EMD on the amplitude function, the HHSA is accomplished via the two-layer EMD of the natural signals and generates the interdependence between the amplitude modulation (AM) frequency and the carrier frequency and a two-dimensional (AM-FM) spectral depiction. FM is the variation of instantaneous frequency over time, while AM is the variation of envelope amplitude over time. The x-axis represents carrier frequency (*f*c), and the y-axis denotes the AM frequency (*f*am). The display of *f*am power of the *f*c results from marginally summed power spectra across time to a specific frequency band (Huang et al., 2016; Juan et al., 2021; Liang et al., 2021; Supplementary Figures 9, 10).

In this study, both the first and second-layer EMDs were performed using an improved complete ensemble EMD with adaptive noise (CEEMDAN; Torres et al., 2011; Colominas et al., 2012, 2014; Liang et al., 2021; Tsai and Liang, 2021) method to attain the first- and second-layer IMFs. Compared to the original or ensemble EMD (Wu and Huang, 2009), the improved CEEMDAN method delivers less mode-mixing, lower reconstruction error (i.e., remaining noise within IMFs), and more consistent frequency distribution ranges in the order of IMFs (Colominas et al., 2014; Tsai and Liang, 2021) for diverse noisy signals. This study’s frequency bins were defined on a dyadic scale (2^–1^, 2^0^, 2^1^, 2^2^, 2^3^, etc.), generating the frequency bands of interest into low-frequency (Lf; 0.5∼1 Hz), delta1 (1∼2 Hz), delta2 (2∼4 Hz), theta (4∼8 Hz), alpha (8∼16 Hz), beta (16∼32 Hz), and gamma (32∼100 Hz). The upper limit of the gamma band 100 Hz is based on the Nyquist rule.

#### 2.2.3. Statistical analysis

**FIGURE 2:**
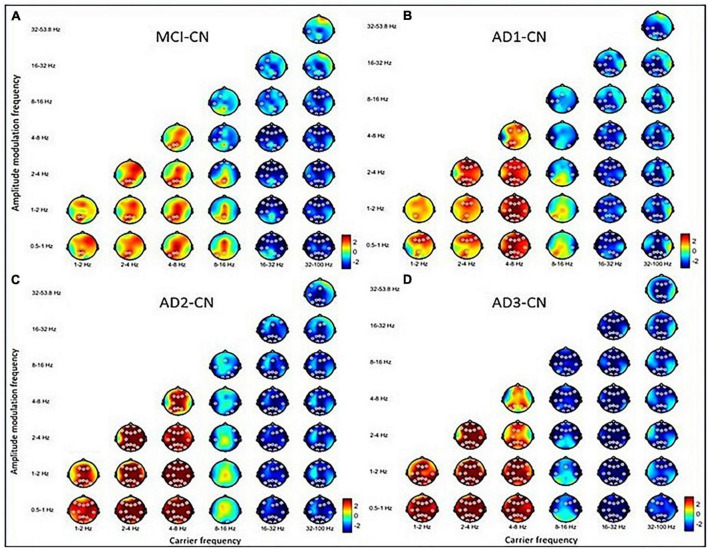
The contrasted HHSA in the eyes-closed (EC) condition for **(A)** MCI–CN, **(B)** AD1-CN, **(C)** AD2-CN, and **(D)** AD3-CN shows increasing AM power of lower-frequency oscillations (low-frequency, delta, and theta bands) with decreasing AM energy in higher-frequency oscillations (beta and gamma bands) across groups. The alpha band acts as a transition zone, revealing increasing AM energy over posterior regions in MCI and AD1 with reducing AM energy over anterior regions across entire patient groups. The x-axis denotes the carrier frequency, while the y-axis denotes AM frequency. The frequency bin is on a dyadic scale (2^– 1^, 2^0^, 2^1^, 2^2^, 2^3^, etc.), except for the gamma band (32–100 Hz). The upper limit of the gamma band is 100 Hz based on Nyquist sampling. The color bar denotes t-statistics ranging from blue (–2) to red (+ 2). White circles indicate that contrast on those EEG channels is statistically significant (*p* < 0.05, two-tailed cluster permutation test).

For statistical comparisons, a two-tailed cluster-based non-parametric permutation (CBnPP) test was conducted (Maris et al., 2007; Groppe et al., 2011) on the multichannel HHSA spectra (channels x AM x frequency bins x carrier frequency bins). The two-dimensional power was processed for the rsEEG signals in the CN, MCI, and AD groups in EC. Pair-wise comparisons were then performed for EC differences between MCI vs. CN, AD1 vs. CN, AD2 vs. CN, and AD3 vs. CN. The neighboring distance between two EEG sensors was defined as 70 mm with 5,000 permutations for each test, which was efficient for multiple comparison problems (Maris and Oostenveld, 2007). Differences between the two groups were analyzed using Student’s *t*-test. All *p*-values were two-tailed, and a *p* < 0.05 was considered significant, which is illustrated as a white circle in the figures (Figures 2, 4).

#### 2.2.4. Classification and prediction based on feature extraction and selection for machine learning

Features from the FFT-, HHT-, and HHSA-based analytical methods were extracted to fit the machine learning algorithms. For the HHSA-based feature extraction, 262,656 features were derived from the topographical whole head map. EEG signatures were extracted from the AM-FM energy map of 19 electrodes, where the individual *f*am, *f*c, or ratio between two EEG signatures were used as features. Subsequently, a correlation was conducted on features to retain one distinct feature from a cluster of features with a correlation higher than 0.95 to remove redundant features. Subsets of 100 features from the tens of thousands of remaining features were applied to the LogitBoost algorithm to select limited (<10) high-ranking important features to avoid overfitting (Guyon and Elisseeff, 2003). These features were then used to fit the machine learning algorithms for binary classification between MCI and AD subgroups and CN. In this study, seven common algorithms were employed, namely, LogitBoost, Bagging (Bag), Gentle adaptive boosting (GentleBoost), Decision tree (Tree), support vector machine (SVM), Naïve Bayes, and K-Nearest Neighbor (K-NN), implemented via the MATLAB software (R2018α, shown in Supplementary Figures 11, 17, 18). Each algorithm underwent 10-fold cross-validation to yield receiver operating characteristic (ROC) curves with area under the ROC curve (AUC) values (Supplementary Figure 11). The performance metrics of sensitivity, specificity, precision, F1 measure, and accuracy are presented in the results (Tables 4, 6; Supplementary Figures 12, 13).

**FIGURE 3:**
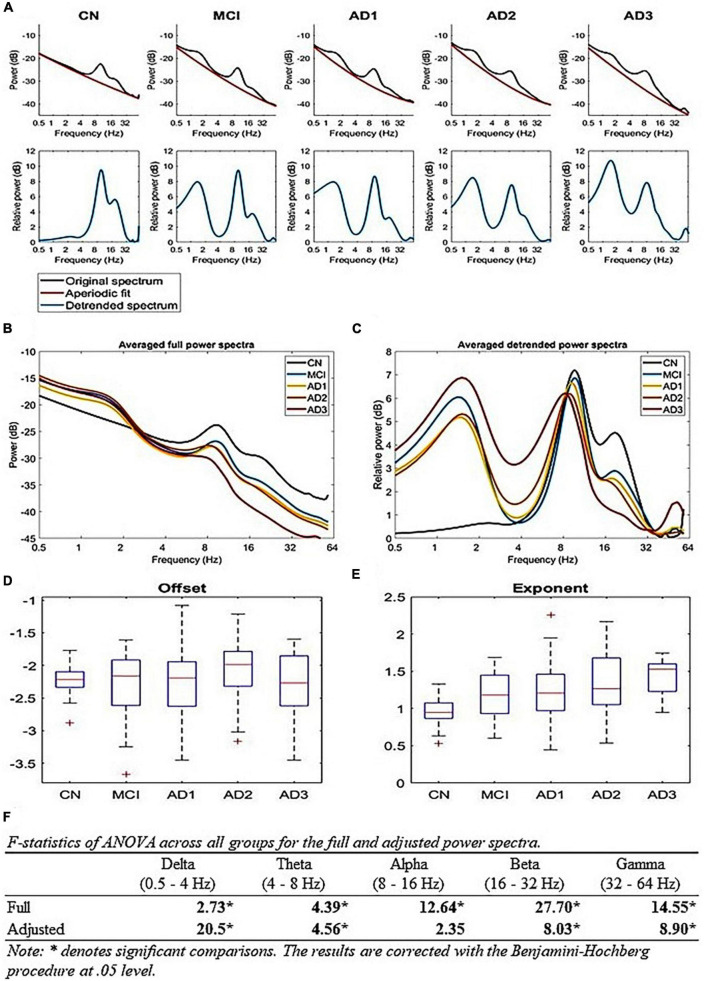
True oscillatory activity using the FOOOF algorithm: **(A)** Periodic and aperiodic fit of EEG data; the upper row illustrates the mean full spectrum (black) and mean aperiodic fit (red) of each group using the FOOOF algorithm. The lower row illustrates the mean periodic component (blue) of each group, **(B,C)** A comparison of full and aperiodic adjusted spectra across all groups, where panel **(B)** is the full mean spectra of all groups and panel **(C)** is the aperiodic adjusted mean spectra of all groups, **(D,E)** Aperiodic parameters across all groups; **(D)** shows that there is no difference for offsets and **(E)** shows that the exponent parameter increases with the progression of AD [*F*_(4,200)_ = 10.83, *p*-value < 1 × 10^– 5^], **(F)** shows the F-statistics of ANOVA across all groups for the full and aperiodic adjusted spectra.

Seventy-two patients in the MCI (CDR = 0.5) cohort were followed up longitudinally with two rsEEG recordings within 3 years. Following clinical labeling, they were further subdivided into MCI-S (*n* = 36) and MCI-C (*n* = 36) subgroups. Subsequently, machine learning algorithms’ performances were evaluated with features derived from different analytical methods to predict AD progression in MCI. The dataset for machine learning was split into training and testing subsets with a 10-fold cross-validation procedure during the model training process. The sequence of our data processing, including carrier frequency and AM was subjected to each decomposition method and FOOOF algorithm, which displayed the periodic and aperiodic components as shown in the in-text Figure 3 and Supplementary Material 15 (Supplementary Figure 20).

## 3. Results

### 3.1. Group-level analysis and contrasted HHSA between AD subgroups and CN subjects

Overall, increasing AM power of LFO became more prominent with disease progression. Decreasing AM power of HFO emerged earlier during MCI, which was sustained along the disease course. Alpha band oscillations functioned as a transitional zone, revealing increasing AM power in posterior regions but reducing AM power in anterior regions in MCI. With disease progression, the alpha band exhibited more widespread decreasing AM power, especially in AD2.

### 3.2. Group-level rsEEG analysis

The three-dimensional (3D) relationship amid the carrier frequency bands, AM frequencies, and topographical energy distribution for MCI vs. CN, AD1 vs. CN, AD2 vs. CN, and AD3 vs. CN in EC is summarized in Figure 2. Energy pattern comparisons between MCI vs. CN and AD1 vs. CN are illustrated following brain regions and classified by topographical electrodes in Tables 2, 3, respectively. Figure 2 is the summary result of the statistical analysis based on CBnPP across the whole AD continuum, whereas Tables 2, 3 display the significant topographical energy patterns between MCI vs. CN and AD1 vs. CN, serving as a comparison reference guide to conventional rsEEG spectral-analysis in MCI and AD patients (Figure 2; Tables 2, 3). Individual group-level comparisons with CN across the AD continuum are illustrated in Supplementary Figures 3–6. Due to age being a confounding factor in this study, we also conducted an analysis of covariance (ANCOVA), which showed consistent results across the AD continuum (Supplementary Figure 21).

**TABLE 2:** MCI vs. CN.

Carrier frequency (FM)		δ 1 (1–2 Hz)		δ 2 (2–4 Hz)			θ (4–8 Hz)				α (8–16 Hz)					β (16–32 Hz)						γ (32–100 Hz)						
**AM**		**L*f***	**δ 1**	**L*f***	**δ 1**	**δ 2**	**L*f***	**δ 1**	**δ 2**	**θ**	**L*f***	**δ 1**	**δ 2**	**θ**	**α**	**L*f***	**δ 1**	**δ 2**	**θ**	**α**	**β**	**L*f***	**δ 1**	**δ 2**	**θ**	**α**	**β**	**γ**
Brain region	Electrode																											
Frontal	FP1																											
	FP2																											
	F7																											
	F3																											
	FZ																											
	F4																											
	F8																											
Central	C3																											
	CZ																											
	C4																											
Parietal	P3																											
	PZ																											
	P4																											
Temporal	T3																											
	T5																											
	T6																											
	T4																											
Occipital	O1																											
	O2																											

The topographical registration of AM frequencies and their corresponding carrier frequency bands displayed a significant increase in delta and theta power density at posterior brain regions. In contrast, decreasing beta and gamma power density was distributed globally. The red color denotes higher energy, while the blue color denotes lower energy in the MCI group. L*f* designates low-frequency oscillation, with the frequency bin 0.5∼1 Hz. The δ1 represents the frequency bin from 1 to 2 Hz, and the δ2 denotes the frequency bin from 2 to 4 Hz. The rest of the frequency bins follow the dyadic scale, as mentioned.

**TABLE 3:** AD1 vs. CN.

Carrier frequency		δ 1 (1–2 Hz)		δ 2 (2–4 Hz)			θ (4–8 Hz)				α (8–16 Hz)					β (16–32 Hz)						γ (32–100 Hz)						
**AM**		**L*f***	**δ 1**	**L*f***	**δ 1**	**δ 2**	**L*f***	**δ 1**	**δ 2**	**θ**	**L*f***	**δ 1**	**δ 2**	**θ**	**α**	**L*f***	**δ 1**	**δ 2**	**θ**	**α**	**β**	**L*f***	**δ 1**	**δ 2**	**θ**	**α**	**β**	**γ**
Region	Electrode																											
Frontal	FP1																											
	FP2																											
	F7																											
	F3																											
	FZ																											
	F4																											
	F8																											
Central	C3																											
	CZ																											
	C4																											
Parietal	P3																											
	PZ																											
	P4																											
Temporal	T3																											
	T5																											
	T6																											
	T4																											
Occipital	O1																											
	O2																											

Topographical registration of AM frequencies with their corresponding carrier frequency bands. Widespread increasing energy density is seen in lower-frequency oscillations, with robust decreases in higher-frequency oscillations. Notably, the increasing power density of the lower-frequency oscillations spreads from posterior to whole brain regions in AD1 when compared with MCI. The annotation is the same as in Table 2.

#### 3.2.1. MCI versus CN

The contrasted HHSA EEG between MCI and CN revealed AM with increasing energy of LFO delta (1∼4 Hz) and theta (4∼8 Hz) bands in posterior brain regions. In contrast, the bands of HFO beta (16∼32 Hz) and gamma (32∼100 Hz) showed decreasing AM power globally. Regarding the interdependency between AM frequency and carrier frequency, the AM-FM energy maps disclosed an increasing power of delta and theta bands modulated with AM frequency below their respective frequency bands. Conversely, the beta and gamma bands’ pronounced decreasing power was modulated with AM frequency below their respective frequencies. The negative modulating pattern of HFO was globally distributed. However, the positive modulating pattern of LFO was scattered over posterior brain regions. The alpha frequency band revealed augmented AM power modulation in low (Lf; 0.5∼1 Hz) and delta frequency in posterior brain regions with attenuated AM power from delta-2 (2∼ 4 Hz) to alpha (8∼16 Hz) frequency in anterior regions (Figure 2A; Table 2; Supplementary Figure 3).

#### 3.2.2. AD1 versus CN

The contrasted HHSA EEG between AD1 and CN showed increasing AM energy of LFO with a reverse trend showing decreasing AM power of HFO globally. The alpha band exhibited decreasing AM energy in frontal and temporal areas, whereas increasing AM energy was only observed in the left temporal area. The AM-FM energy maps depicted densely augmented LFO power with AM frequency below their respective frequencies. Inversely, the prominent attenuated energy of HFO bands was modulated by AM frequency below the beta and gamma bands, respectively. The negative modulating trend of the HFO was widely distributed. Meanwhile, the positive modulating trend of the LFO spread globally in delta-2 and theta bands but sparsely in the delta-1 band (Figure 2B; Table 3; Supplementary Figure 4).

#### 3.2.3. AD2 versus CN

The contrasted HHSA EEG between AD2 and CN depicted increasing AM power of LFO with decreasing AM energy of HFO globally. Declining AM power of the alpha band was found in frontal, temporal, and occipital regions, a reverse brain oscillatory pattern compared with MCI. The AM-FM energy maps showed increasing power of LFO bands. Contrarily, HFO bands manifested decreased power. The negative modulating trend in the energy density of HFO was globally distributed. Meanwhile, the positive modulating trend of LFO was densely dispersed over the whole brain. The energy map of the alpha band in the frontal, central, temporal, and occipital areas showed a sparse decreasing power density pattern (Figure 2C; Supplementary Figure 5).

#### 3.2.4. AD3 versus CN

The contrasted HHSA between AD3 and CN displayed increased AM energy of LFO globally. Meanwhile, decreasing AM power of alpha, beta, and gamma bands was widely dispersed. The AM-FM energy maps revealed markedly augmented LFO power modulated with AM frequency below their respective frequencies. Conversely, attenuated alpha and HFO energy showed AM frequency modulation below alpha, beta, and gamma bands. Modulating trends in the energy density were broadly distributed except for the theta band, which was restricted to the parietal region of the delta-modulating theta frequency band (Figure 2D; Supplementary Figure 6).

### 3.3. Classification performance of extracted features for best-fitted algorithms

The performance of seven binary classification algorithms, namely, LogitBoost, Bag, GentleBoost, Decision Tree, Support Vector Machine (SVM), Naïve Bayes, and k –nearest neighbor (k-NN), utilized features extracted from three analytical methods, encompassing HHSA, HHT, and windowed FFT deployed to classify MCI or CN, as shown in Table 4 (Supplementary Figure 12). HHSA-based feature extraction outperformed other methods, with the GentleBoost algorithm surpassing other classifiers with a sensitivity of 82%, specificity of 80%, and accuracy of 81% (Table 4 and Supplementary Figure 19). Therefore, only the performance summary of the best algorithm deployed in group-level analyses utilizing HHSA-extracted features is presented in Table 5. Regarding HHSA-based binary classification between MCI vs. CN, AD1 vs. CN, AD2 vs. CN, and AD3 vs. CN, five, six, four, and three features best fitted the classifiers, respectively. The Bag algorithm outperformed other classifiers in the binary classification for AD1 vs. CN and AD2 vs. CN. SVM and Naïve Bayes transcended other classifiers for AD3 vs. CN (Table 5). HHSA-extracted features contained channel location, *f*am, and *f*c. Distribution of carrier frequencies in LFO (delta and theta bands) vs. HFO (alpha, beta, and gamma bands) for binary classification between MCI and CN, as well as AD1 and CN, were 2 vs. 3 and 2 vs. 4. The best-fitted features in binary classification between moderate AD and CN and severe AD and CN were based on a feature ratio. The feature ratios were LFO/LFO, HFO/HFO, HFO/LFO, and HFO/LFO in the binary classification between AD2 and CN, whereas they were HFO/LFO, LFO/HFO, and HFO/HFO in the binary classification between AD3 and CN (Table 5).

**TABLE 4:** Performance evaluation of classification algorithms deployed to discriminate between MCI and CN used features extracted from group-level comparison by different signal analytical methods.

		LogitBoost	Bag	GentleBoost	Tree	SVM	Naive	k-NN
HHSA	Sensitivity	0.71	0.65	0.82	0.71	0.59	0.82	0.59
	Specificity	0.80	0.85	0.80	0.75	0.80	0.70	0.80
	Precision	0.75	0.79	0.78	0.71	0.71	0.70	0.71
	F1-measure	0.73	0.71	0.80	0.71	0.65	0.76	0.65
	Accuracy	0.76	0.76	0.81	0.73	0.70	0.76	0.70
HHT	Sensitivity	0.35	0.41	0.41	0.47	0.53	0.71	0.29
	Specificity	0.55	0.65	0.55	0.50	0.60	0.35	0.35
	Precision	0.40	0.50	0.44	0.44	0.53	0.48	0.28
	F1-measure	0.38	0.45	0.42	0.46	0.53	0.57	0.29
	Accuracy	0.46	0.54	0.49	0.49	0.57	0.51	0.32
FFT	Sensitivity	0.71	0.71	0.71	0.65	0.71	0.82	0.53
	Specificity	0.80	0.65	0.75	0.70	0.75	0.75	0.85
	Precision	0.75	0.63	0.71	0.65	0.71	0.74	0.75
	F1-measure	0.73	0.67	0.71	0.65	0.71	0.78	0.62
	Accuracy	0.76	0.68	0.73	0.68	0.73	0.78	0.70

**TABLE 5:** The performance summary of the best classifiers deployed in group-level comparisons used HHSA-based feature extraction; fam denotes amplitude modulation frequency, fc represents carrier frequency, and SVM denotes support vector machine.

Classification	Performance			Algorithm	Features (n)	Features/Feature ratio	
	**Sensitivity**	**Specificity**	**Accuracy**			**Numerator**	**Denominator**
MCI vs. CN	0.82	0.80	0.81	Gentle Boost	5	Fp1, *f*am 0–1 Hz, fc 16–32 Hz F4, *f*am 2–4 Hz, *f*c 4–8 Hz F8, *f*am 1–2 Hz, *f*c 2–4 Hz T6, *f*am 0–1 Hz, *f*c 32–64 Hz O2, *f*am 4–8 Hz, *f*c 16–32 Hz	
AD1 vs. CN	0.94	0.80	0.86	Bag	6	Fp1, *f*am 0–1 Hz, *f*c 16–32 Hz F4, *f*am 0–1 Hz, *f*c 32–64 Hz F4, *f*am 1–2 Hz, *f*c 1–2 Hz Fz, *f*am 0–1 Hz, *f*c 32–64 Hz T3, *f*am 8–16 Hz, *f*c 8–16 Hz P4, *f*am 4–8 Hz, *f*c 4–8 Hz	
AD2 vs. CN	0.93	0.90	0.91	Bag	4	Fp1, *f*am 0–1 Hz, *f*c 4–8 Hz Fp2, *f*am 0–1 Hz, *f*c 32–64 Hz Fz, *f*am 0–1 Hz, *f*c 32–64 Hz P4, *f*am 8–16 Hz, *f*c 8–16 Hz	Fp2, *f*am 0–1 Hz, *f*c 1–2 Hz Fp2, *f*am 1–2 Hz, *f*c 32–64 Hz F4, *f*am 0–1 Hz, *f*c 2–4 Hz P3, *f*am 1–2 Hz, *f*c 4–8 Hz
AD3 vs. CN	0.75	1.0	0.96	SVM and Naïve Bayes	3	P3, *f*am 32–64 Hz, *f*c 32–64 Hz P4, *f*am 4–8 Hz, *f*c 4–8 Hz O2, *f*am 4–8 Hz, *f*c 8–16 Hz	F8, *f*am 1–2 Hz, *f*c 2–4 Hz P4, *f*am 8–16 Hz, *f*c 8–16 Hz P4, *f*am 1–2 Hz, *f*c 16–32 Hz

### 3.4. Longitudinal follow-up differences in HHSA-rs EEG between MCI-C and MCI-S

**FIGURE 4:**
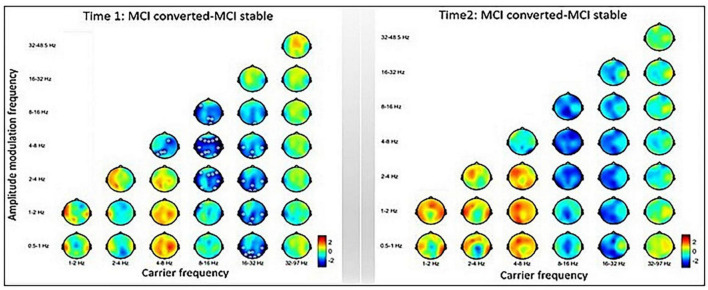
Inter-group comparisons between MCI-converted and MCI-stable subgroups. **Left:** The contrasted HHSA between MCI-C and MCI-S subgroups of time 1 (baseline) shows decreasing AM power of the alpha band over the frontal, temporal, and parietal brain regions. Meanwhile, reduced AM energy is seen in the beta band over the temporal, parietal, and occipital brain regions. **Right:** The HHSA contrast of time 2 (3 years apart) shows a trend of increasing AM power of the delta and theta bands coupled with decreasing AM power of the alpha and beta bands. However, no statistically significant contrast is seen between electrodes. The annotation is the same as Figure 2 [*p* < 0.05, CBnPP with 5,000 permutations, two-tailed; max distance (cluster) = 70 mm].

The inter-group comparisons between MCI-C and MCI-S are displayed in Figure 4. The left panel shows the baseline-contrasted HHSA between MCI-C and MCI-S (i.e., Time 1), exhibiting decreasing AM power from theta to delta2 (2∼4 Hz) band at the alpha *f*c in the frontal, temporal, and parietal regions. In the temporal, parietal, and occipital regions, decreasing AM power is seen from alpha to low-frequency (0.5∼1 Hz) bands at the beta *f*c. However, following the Bedrosian theorem (Bedrosian, 1963), diminished AM power of theta frequency band modulating theta carrier frequency should not be considered a reliable signature. The right panel shows the contrasted HHSA between MCI-C and MCI-S at a 3-year follow-up time point (i.e., Time 2), exhibiting no statistically significant difference. However, a trend of decreasing AM power in alpha and beta bands, accompanied by increasing AM power in delta and theta bands, is seen (Figure 4).

#### 3.4.1. Intra-group comparisons in longitudinal HHSA-rsEEG of MCI-C and MCI-S

The intra-group comparisons within the 3-year longitudinal follow-up rsEEG in MCI-C and MCI-S subgroups are shown in Supplementary Figure 7. The left panel displays that MCI-C within-group analysis shows a trend of increasing AM power in alpha and beta fc, with decreasing AM power in theta and delta fc. The right panel reveals that the MCI-S within-group analysis shows a trend of increasing AM energy in the alpha fc. However, no statistically significant contrasts are seen within groups (Supplementary Figure 7).

#### 3.4.2. Performance evaluation of extracted features by various analytical methods

The performance of seven binary classification algorithms applied to discriminate between MCI-S and MCI-C, with their features extracted from baseline rsEEG contrast between subgroups using the HHSA, HHT, and windowed FFT, is shown in Table 6 (Supplementary Figure 13). Features differentiating MCI-C from MCI-S in baseline rsEEG could function as predictors for progression in MCI. Regarding the sensitivity, SVM (90%) yielded the best results for HHSA, Tree (70%) yielded the best results for HHT, whereas LogitBoost (80%), Tree (80%), and K-NN (80%) yielded the best results for the windowed FFT-based features extraction, respectively. Meanwhile, concerning specificity, Tree (80%), SVM (70%), and Bag (70%) reported the best performance by HHSA, HHT, and windowed FFT-based features extraction, respectively (Table 6).

**TABLE 6:** Performance evaluation of prediction algorithms used features extracted from baseline rsEEG comparison between MCI-C and MCI-S subgroups by different analytical methods.

		LogitBoost	Bag	GentleBoost	Tree	SVM	Naive	k-NN
HHSA	Sensitivity	0.50	0.50	0.70	0.70	0.90	0.70	0.50
	Specificity	0.70	0.70	0.60	0.80	0.50	0.60	0.50
	Precision	0.63	0.63	0.64	0.78	0.64	0.64	0.50
	F1-measure	0.56	0.56	0.67	0.74	0.75	0.67	0.50
	Accuracy	0.60	0.60	0.65	0.75	0.70	0.65	0.50
HHT	Sensitivity	0.60	0.60	0.60	0.70	0.50	0.30	0.50
	Specificity	0.30	0.50	0.50	0.50	0.70	0.50	0.60
	Precision	0.46	0.55	0.55	0.58	0.63	0.38	0.56
	F1-measure	0.52	0.57	0.57	0.64	0.56	0.33	0.53
	Accuracy	0.45	0.55	0.55	0.60	0.60	0.40	0.55
FFT	Sensitivity	0.80	0.60	0.60	0.80	0.50	0.50	0.80
	Specificity	0.50	0.70	0.50	0.60	0.60	0.50	0.60
	Precision	0.62	0.67	0.55	0.67	0.56	0.50	0.67
	F1-measure	0.70	0.63	0.57	0.73	0.53	0.50	0.73
	Accuracy	0.65	0.65	0.55	0.70	0.55	0.50	0.70

HHT and windowed FFT-based features contained channel location and *f*c. Concurrently, HHSA-based feature extraction added an *f*am dimension. The distribution of *f*c in LFO (delta and theta bands) vs. HFO (alpha, beta, and gamma bands) in HHSA-extracted, HHT-extracted, and FFT-extracted features were 4 vs. 3, 6 vs. 1, and 3 vs. 4 (Supplementary Figure 8).

### 3.5. Conversion rate of Alzheimer’s clinical syndrome in MCI patients

The conversion rate of MCI to AD in the 3-year longitudinal follow-up yielded a percentage of 17% within 1 year, 36% in 2 years, and 44% in 3 years following clinical diagnostic procedures. This is shown in Table 7.

**TABLE 7:** Probability of 3-year longitudinal follow-up of MCI progressing to mild AD yearly.

MCI patients with CDR 0.5		
	**MCI-stable** **(MCI-S)**	**MCI-converted** **(MCI-C)**
*n*	36	36
Conversion to mild AD within 1 year	0	16.6% (*n* = 6)
Conversion to mild AD within 2 years	0	36.1% (*n* = 13)
Conversion to mild AD within 3 years	0	44.4% (*n* = 16)

## 4. Discussion

The HHSA provides whole-head topography contrasts following AM-FM interdependency orientation, where these spectral representations are capable of extracting the interdependence of *f*am and *f*c for further utilization to examine CFC using the standard PAC method (Canolty and Knight, 2010) to depict potential local and long-range CFCs (Juan et al., 2021; Liang et al., 2021). The presence of AM power in the *f*c is the necessary condition for CFC though CFC requires identifying the modulation source (Juan et al., 2021). The HHSA results are collapsed across time as the time variation in rsEEG is less important than the overall AM-FM structure. The advantage of EMD-based HHSA over FFT and wavelet transform is that EMD is apt for analyzing non-stationary and non-linear data, which is not feasible when applying conventional FFT-based spectral analysis (Huang et al., 1998, 2016). The power spectrum consists of periodic and aperiodic components, where the differential effect of both components has been demonstrated in physiological and pathological aging (He, 2014; Donoghue et al., 2020; van Nifterick et al., 2023). Thus, we used the multitaper-based power spectra of each group to fit the model and adjust the aperiodic component, which yields the periodic component and reveals oscillatory activities in LFO and HFO (Figure 3). The AM-FM energy map genuinely displays systemic fluctuating patterns across the AD spectrum, which has yet to be reported. Fitting our data to the FOOOF algorithm yields aperiodic and periodic components compared with recently published research. This is further discussed in the novel alpha and gamma AM findings. Finally, regarding machine learning, adding an AM dimension boosts their performance for classification and prediction.

### 4.1. Converging findings of altered brain oscillations in MCI and the AD continuum

#### 4.1.1. Altered spectral power in carrier frequency across the AD continuum correlates with neurobiological biomarkers

The typical pattern of spectral analysis of rsEEG in MCI and AD revealed augmented power of delta and theta bands with attenuated power of alpha and beta bands, accompanied by slowing of the alpha peak frequency, also known as EEG slowing (Supplementary Figure 14; Dauwels et al., 2010; Hsiao et al., 2013; van Straaten et al., 2014; Wu et al., 2014; Babiloni et al., 2018). In this study, supporting evidence is provided for the characteristic brain oscillatory patterns of MCI and AD, derived from various measurements with conventional FFT-based spectral analysis of rsEEG and advanced analysis of spectral power fluctuation over time (i.e., amplitude modulation; Figure 2). In addition, integrating the evidence of neurobiological profiles across the AD spectrum (Jack et al., 2018) with brain oscillatory signatures provides a non-invasive, affordable, and reproducible surrogate marker for screening, detecting, and follow-up in large populations with early cognitive decline (Maestú et al., 2019).

Our results showed an increment in AM power of LFO (delta and theta bands) with disease progression, accompanied by a decrement in AM power of HFO (beta and gamma bands) in MCI patients, sustained along the disease course. Incorporating grading of neurobiological profiles in the AD continuum (Jack et al., 2018) and correlation with EEG signatures paved a broad way for rsEEG utility in clinical applications (Maestú et al., 2019). For instance, correlating global field power (GFP; Lehmann and Skrandies, 1980) and CSF profiles revealed that the CSF amyloid-beta 42 effect correlates with GFP augmentation of delta and theta, whereas CSF p-tau and t-tau effects were associated with GFP attenuation of alpha and beta bands (Smailovic et al., 2018). MCI patients display increased prefrontal and occipital delta power, which is negatively correlated with cognitive function, structural MRI, and AD-pattern FDG-PET (Babiloni et al., 2018; Nakamura et al., 2018), while increased global theta power negatively correlates with memory capacity, structural MRI, and cognitive performance (Musaeus et al., 2018a; Nakamura et al., 2018). Notably, increased global theta power may not be a characteristic marker of MCI caused by AD (Nakamura et al., 2018). In our results, MCI patients exhibited increased AM power of delta and theta bands in posterior brain regions and decreased beta and gamma AM power globally compared with CN (Figure 2A; Table 2; Supplementary Figure 3). Apart from findings in the spectral power of carrier frequency, we also report AM power of dual-pattern alpha oscillations and global attenuated gamma oscillations, which depict full-band spectro-temporal representations of rsEEG signals in MCI (Cassani and Falk, 2019).

In the AD continuum, our results disclosed globally augmented AM power of LFO. In contrast, there was widespread attenuated AM power of HFO with sparsely increasing posterior alpha AM power in AD1 and pronounced decreasing alpha AM power in AD2 (Figures 2B, C; Supplementary Figures 3, 4). Regarding the spatial source dipole, alpha and beta source activities shifted anteriorly in AD1 patients with increased delta and theta GFP and decreased alpha GFP, which have been deployed in linear discriminant analyses, yielding the best performance in binary classification (Huang et al., 2000). Associating rsEEG with neurobiological biomarkers may make clinical applications plausible. The increasing delta source activity was positively correlated with cortical hypometabolism of FDG-PET in AD-pattern areas and cognitive decline (Babiloni et al., 2016). Decreasing delta and beta GFP in AD1 was also correlated with CSF p-tau and t-tau levels (Smailovic et al., 2018). Furthermore, global augmented theta power was correlated with cognitive deficit and total tau level in AD1 (Musaeus et al., 2018a). Across the CN to MCI to AD continuum in our results (Figure 2), augmented AM power of delta and theta bands extending anteriorly with attenuated posterior alpha band AM power, accompanied by global attenuated HFO AM power, reflects disease advancement via increasing delta and theta AM power with decreasing alpha and beta AM power (Fraga et al., 2013).

#### 4.1.2. AM modulating trend is determined by the spectral pattern of carrier frequency in the AD continuum

Current studies show spectral patterns in MCI and AD but lack systematic reports of AM dimension’s characteristics in AD, which is the gist of this study. The AM power shown here is comparable to band-limited power (BLP), which extracts AM power based on FFT band-passed filter followed by the Hilbert transform. Fraga and Falk’s reports encompass a similar method to our study by analyzing AM power to discriminate between AD1 and CN as well as AD1 and AD2 (Falk et al., 2012; Fraga et al., 2013). In contrast, we used EMD-based spectral analysis with subsequent processing of extracted envelopes by secondary EMD to generate corresponding AM domains (Huang et al., 2016). Their findings in AD1 with attenuated AM power of alpha and beta bands and augmented LFO AM power are consistent with our results (Figure 2B; Table 3; Supplementary Figure 4); both portray an AM power-modulating trend following the spectral pattern of carrier frequency across the AD continuum or variation of AM power following the EEG slowing pattern with AD progression (Falk et al., 2012; Fraga et al., 2013; Figure 2; Tables 2, 3). Regarding the AM-FM relationship (Tables 2, 3), lower frequency amplitude modulation of HFO is more important than amplitude modulation of LFO when discriminating MCI from CN. Meanwhile, differentiating AD1 from CN, lower frequency amplitude modulation of HFO (alpha, beta, and two gammas) is more essential than the amplitude modulation of LFO (Table 5).

#### 4.1.3. Parameterizing spectral power of carrier frequency (fc) and corresponding AM into aperiodic and periodic components

In our results, the interdependency between AM frequency and *f*c behaves like a hierarchical architecture between oscillations, where the phase of slower oscillations modulates the amplitude of faster oscillations (Lakatos et al., 2005). In a monkey’s auditory passive listening task in the awake condition, brain waves showed that the delta phase modulates theta amplitude, while the theta phase modulates the gamma amplitude, which is similar to our frequency architecture in the AM-FM energy map. Amplitude modulation occurs when the amplitude of a carrier signal (*f*c) varies in proportion to the message signal. At the same time, the frequency and phase are kept constant. Phase coupling synchronizes two or more rhythmic or oscillating processes with a fixed phase difference. However, the modulation source may come from the effect of the neurotransmitter (Pepeu et al., 2015), perceptual entrainment (Fiebelkorn and Kastner, 2018), local or long-range cross-frequency coupling (Liang et al., 2021), or body rhythm (Klimesch, 2018).

To examine whether our extracted brain oscillations contain any true oscillatory activity, we applied the FOOOF algorithm (Donoghue et al., 2020) to decipher *f*c and AM power spectra into aperiodic and periodic components (Figure 3A). Since the relevant analysis for EMD-based decomposition is not applicable, we used multitaper time-frequency decomposition to generate the respective power spectra (Figure 3B). The results show true alpha-beta oscillatory activity (8–32 Hz) across all groups with LFO activity (below 8 Hz, including delta and theta bands) for MCI and AD continuum (Figure 3C). Aperiodic activities primarily contribute to the gamma activity. Similar to the HHSA, the adjusted lower-frequency power increases, whereas the beta power decreases with AD progression (Figure 3F). There is no difference in the adjusted alpha power. The gamma power in AD3 is larger than in other groups (Figure 3C). However, the result may need to be more stable given the small sample size (16 participants). On the other hand, the aperiodic exponent also increases with AD progression [Figure 3E; *F*(4,200) = 10.83, *p*-value < 10-5]. The overall results show that the EEG slowing is contributed to by the increasing aperiodic exponent along with the increment of periodic LFO and decrement of HFO. Similarly, the FOOOF analysis on gamma (IMFs 1 and 2), beta (IMF 3), and alpha (IMF 4) AM yields both periodic (Supplementary Figure 15, middle column) and aperiodic components (Supplementary Figure 15, right column). The periodic spectra of gamma, beta, and alpha AM still exist in oscillatory activities ranging from 2 to 32 Hz, 2 to 32 Hz, and 1 to 16 Hz, respectively, with center frequencies at 8 Hz, 3 Hz, and 3 Hz (Supplementary Figure 15, middle column).

#### 4.1.4. Alpha AM spectral pattern in MCI and AD- alpha transitional or fulcrum hypothesis

Our results illustrate an anterior-posterior spatial-specific alpha AM pattern, revealing augmented alpha AM power in posterior and attenuated alpha AM power in anterior regions (Supplementary Figure 3; Table 2). Previous studies demonstrated attenuated posterior alpha source and decreased alpha-band-associated functional connectivity of rsEEG in MCI (Babiloni et al., 2006c,d, 2018). However, some rsEEG studies also reported augmented alpha spectral power in MCI (Huang et al., 2000; Babiloni et al., 2006b; Moretti et al., 2009; Caravaglios et al., 2015). These studies reveal a discrepancy in the alpha frequency bin. In our study, the alpha range was 8∼16 Hz and was determined by dyadic filter-bank properties of IMF (Flandrin et al., 2004), encompassing Huang’s alpha (8–11.5 Hz), Babiloni’s alpha2 (10.5–13 Hz), and Caravaglios’s alpha 3 (9.62–11.62 Hz). Therefore, our increased posterior alpha AM power phenomenon in MCI is consistent with augmented high alpha which is seen in the abovementioned studies (Babiloni et al., 2006b; Caravaglios et al., 2015; Moretti et al., 2017). This reflects a spatial-specific anterior-posterior dual pattern of alpha AM oscillations in our study.

Mild cognitive impairment is a cognitively transitional stage between CN and mild AD, supported by neurophysiological signals (Babiloni et al., 2006b). Our results unveiled evolving brain oscillation changes from MCI to AD continuum, with reciprocal oscillatory patterns between LFO and HFO (Figure 2). Specifically, the alpha oscillations may be a transitional phenomenon that portrays progressively altered brain oscillations between MCI and mild AD. In previous studies, comparisons between CN, MCI, and mild AD revealed progressively attenuated alpha1 (8∼10.5 Hz) source activity, with MCI showing augmentation in alpha2 (10.5∼13 Hz; Babiloni et al., 2006b). This insinuates a neurophysiological compensation for early cognitive decline (Huang et al., 2000; Babiloni et al., 2006b; Dubois et al., 2018). Posterior alpha rhythm is spawned by the ensemble synchronization of oscillatory activities between the thalamocortical loop and cortico-cortical interactions, mediating transmission of sensorimotor information between the subcortical and cortical loop, as well as retrieval of stored memory (Steriade and Timofeev, 2003; Klimesch et al., 2007). Thus, the high alpha power in rsEEG indicates a healthy aging brain (Babiloni et al., 2006e). Our study showed gradually attenuated anterior alpha AM power from MCI to mild AD, suggesting a progressive cholinergic deficit due to basal forebrain dysfunction advancing to degeneration in MCI and early AD, respectively (Schliebs and Arendt, 2011; Pepeu et al., 2015). Meanwhile, augmented posterior alpha AM power suggests an intact thalamocortical and cortico-cortical loop that compensates for MCI in the non-demented elderly (Moretti et al., 2009; Dubois et al., 2018).

Alternatively, the alpha band may serve as a fulcrum to explain the AM power-changing pattern between LFO and HFO, which is likely a manifestation of a rotational shift in the 1/f-like background activity of the brain rhythm and may be aperiodic in nature. The effects of the aperiodic exponent upon *fc* power is further discussed in supplementary materials (Supplementary Figures 22, 23). Nonetheless, the multitaper applied in rsEEG deals with the unadjusted full spectra of AM power (Supplementary Figure 15, left column), whereas the Detrended Fluctuation analysis (DFA; Linkenkaer-Hansen et al., 2001) and FOOOF algorithm can handle the aperiodic component (Donoghue et al., 2020; Supplementary Figure 15, right column). According to Donoghue et al. (2020), brain oscillations contain periodic and aperiodic components and demonstrate the implications of the aperiodic component’s effect on the interpretation of canonical narrowband frequency analysis and age effect on both components (Donoghue et al., 2020). The age effect on periodic power spectra consists of decreased center frequency, adjusted alpha power, and a reduced offset and exponent in the aperiodic component (Donoghue et al., 2020). However, a recent study showed that adjusted alpha power reveals no statistically significant difference between younger and older adults (Merkin et al., 2023), which means an aperiodic component contributes to the unadjusted alpha power difference. In our data, *f*c spectral power fitted in the FOOOF algorithm reveals an increased aperiodic exponent (Figure 3E) and shift in alpha peak frequency (Figure 3C) without a difference in adjusted alpha power (Figure 3F). Similarly, alpha AM power spectra fitted in the FOOOF algorithm yield periodic components with two peak frequencies at 3 and 8 Hz and similar aperiodic exponents of all groups (Supplementary Figure 15, middle and right column). As Wang et al. (2023) revealed in the adjusted power spectrum, the alpha periodic component revealed decreased power in AD compared to CN, with an increased aperiodic offset and exponent (Wang et al., 2023). The changing trend in the center frequency is consistent in the aging and AD process. In contrast, the trend in aperiodic parameters (i.e., offset and exponent) is the opposite in both conditions, decreases across aging (Donoghue et al., 2020; Merkin et al., 2023) and increases in AD (Figure 3E; Wang et al., 2023). If the results in our data and Wang et al. (2023) are reliable, then the alpha band is likely a fulcrum balancing the lower-frequency (LFO; delta and theta) power and higher-frequency (HFO; beta and gamma) power. The result shows increased aperiodic exponent in AD patients, which means the slope is steeper, leading to increasing LFO power and decreasing HFO power (Figure 3C; Wang et al., 2023). In terms of excitation/inhibition (E/I) imbalance, it depicts a hypoexcitability state (Gao et al., 2017), which is not the case in the AD animal model of Palop and Mucke (2016), which is also contrary to a recent study showing that AD patients have a lower aperiodic gamma frequency exponent and decreased DFA exponent, which suggests a flatter slope (i.e., hyperexcitability state) and an E/I imbalanced state, respectively (van Nifterick et al., 2023). Due to inconsistent results around this issue, more research on further analytical methods to answer this hypothesis is needed, which is also a good direction for future work, essentially combining HHSA and FOOOF methods to fully display the nature of rsEEG (Figure 3; Supplementary Figure 15).

#### 4.1.5. Decreased AM power of alpha and beta oscillations predict AD progression

In our 3-year longitudinal follow-up dataset, baseline rsEEG signals of MCI discriminated between MCI-C and MCI-S. Specifically, MCI-C exhibited lower alpha and beta band AM power over widespread posterior brain regions compared to MCI-S (Figure 4). Similar results were shown by Huang et al. (2000), who longitudinally followed up on an MCI group for approximately 2 years. Baseline EEG comparisons revealed that the MCI-C subgroup exhibited decreasing alpha GFP. In other words, compared with AD, MCI-S showed increasing GFP of alpha (8∼11.5 Hz) and beta2 (16∼19.5 Hz) bands with source dipoles in posterior brain regions (Huang et al., 2000). In an FFT-based spectral analysis, inter-group comparisons at baseline for MCI and mild AD disclosed that posterior-channel alpha power (8∼11.5 Hz) was positively correlated with cognitive function and could discriminate between MCI-S and MCI-C, as well as MCI-S and mild AD (Luckhaus et al., 2008). Yet, another 1.9-year longitudinal follow-up study unveiled that combining six EEG-based biomarkers outputs the best classification performance between MCI-S and MCI-C, which is relevant to decreasing alpha and beta band power (Poil et al., 2013). Additionally, attenuated parietal beta1 (13∼17.99 Hz) power predicted disease progression in MCI-C (Musaeus et al., 2018b). Indeed, our results (Figure 4) disclosed an increased global alpha (8∼16 Hz) AM power and posterior beta (16∼32 Hz) AM power in MCI-S compared with MCI-C, concurring with the aforementioned studies and suggests a compensatory phenomenon for early cognitive decline with resistance toward progression to AD (Moretti et al., 2009; Dubois et al., 2018). Contrariwise, attenuated alpha and beta AM power in MCI can be predictive markers for progression to AD (Figure 4).

#### 4.1.6. Decreased gamma AM power in MCI and the AD continuum

Due to methodological limitations, previous studies rarely dealt with brain oscillations beyond 30 Hz in MCI or AD (Supplementary Figure 16). The altered gamma oscillatory pattern in MCI and AD remains unclarified. Our results unveiled decreasing global AM power of gamma oscillations in MCI that was sustained along the disease course. A comparison of multitaper and HHT-based averaged carrier frequency power spectra of all groups (Supplementary Figure 16, left panel) shows multitaper-based power spectra (blue curve) following a power law with a center frequency of 9 Hz. In contrast, HHT-based power spectra (red curve) display a flatter curve with oscillatory activity ranging from 4 to 56 Hz. Much energy is retained in the higher frequency range, including the gamma range. Regarding the comparison of multitaper and HHT-based averaged gamma AM power spectra of all groups (Supplementary Figure 16, right panel), multitaper-based spectra (blue curve) still follow a power law with a center frequency of 9 Hz. Contrarily, HHT-based spectra (red curve) exhibit a flatter curve preserving much power in the gamma range, which might attribute to theta or alpha amplitude modulation of gamma power (Osipova et al., 2008; Friese et al., 2013). In summary, HHT is an EMD-based adaptive signal analysis that is able to retain non-linear components and gain a high signal-to-noise ratio in complex signals, such as EEG analysis (Huang et al., 1998, 2016; Quinn et al., 2021). Multitaper-based averaged carrier frequency power spectra of all groups are fitted in the FOOOF algorithm; adjusted power spectra reveal that the gamma power of the AD3 group is larger than other groups (Figures 3C, F). However, given the small sample size, the result may need to be more stable after collecting more data. Multitaper-based gamma AM power spectra, fitted in the FOOOF analysis, show oscillatory activities ranging from 2 to 32 Hz with a center frequency of 7–8 Hz and increased aperiodic exponents in MCI, AD1, and AD2 as well as decreased aperiodic offset with AD progression (Supplementary Figure 15, middle and right column).

Gamma oscillations represent cluster neuronal synchronization in cortical activation (Moruzzi and Magoun, 1949; Munk et al., 1996) related to perception (Llinás and Ribary, 1993; Engel et al., 1999), short-term memory (Tallon-Baudry and Bertrand, 1999), and attention (Tiitinen et al., 1993; Fries et al., 2001). These oscillations ensue from the interneuron network gamma (ING), pyramidal interneuron network gamma (PING), and persistent gamma activity (Whittington et al., 2011). Transgenic mice models of AD exhibit attenuated gamma oscillations, likely due to the formation of increasing amyloid-beta (Aβ) plaque within subcortical regions and later on in cortical regions with disease progression. Iaccarino et al. (2016) showed that behaviorally reduced driven gamma oscillations before the onset of plaque formation or cognitive decline in an AD mouse model could be modulated by inducing genes associated with the morphological transformation of microglia via optogenetically driving fast-spiking parvalbumin-positive (FS-PV)-interneurons at gamma (40 Hz) range to mitigate AD pathology (Iaccarino et al., 2016). In our study (Figure 2), attenuated AM of gamma oscillations over widespread brain regions from MCI to late-stage AD likely reflects the underlying GABAergic interneuron dysfunction or GABAergic neuronal loss. The AD mice model’s findings support our results and motivate future gamma entrainment using sensory stimulation or other non-invasive brain-stimulating interventions to prevent or attenuate disease progression (Reinhart and Nguyen, 2019; Adaikkan and Tsai, 2020; Grover et al., 2021).

### 4.2. Machine learning issue in features selection

#### 4.2.1. Discrepancy in variables between group and individual level analysis

The features selected were either a single feature or a feature ratio. Each feature code included channel name, fam, and fc, e.g., Fp1 (channel name), fam 0∼1 Hz, and fc 16∼32 Hz. The best-fitted five features deployed in the Gentle Boost classifier for discriminating between MCI and CN (Table 5) follow the colored squares in the AM-FM power maps (Table 2): [FP1 (frontal), fam 0∼1 Hz (Lf, low-frequency), and fc 16∼32 Hz (beta)], [F4 (frontal), fam 2∼4 Hz (delta2), and fc 4∼8 Hz (theta)], [F8 (frontal), fam 1∼2 Hz (delta1), and fc 2∼4 Hz (delta2)], [T6 (temporal), fam 0∼1 Hz (Lf), and fc 32∼64 Hz (gamma)], [O2 (occipital), fam 4∼8 Hz (theta), and fc 16∼32 Hz (beta)]. Notably, the second (F4 channel) and fourth (F8 channel) features are inconsistent with the AM-FM power map. However, the feature in F8 abuts the colored squares, in which some matched features might be deleted during the dimensionality reduction process (Agarwal, 2019). The best-fitted six features deployed in the Bag classifier for differentiating between AD1 and CN (Table 5) follow the colored squares in the AM-FM power maps in Table 3: [FP1 (frontal), *f*am 0∼1 Hz (Lf), and *f*c 16∼32 Hz (beta)], [F4 (frontal), fam 0∼1 Hz (Lf), and *f*c 32∼64 Hz (gamma)], [F4 (frontal), *f*am 1∼2 Hz (delta1), and *f*c 1∼2 Hz (delta1)], [Fz (frontal), fam 0∼1 Hz (Lf), and fc 32∼64 Hz (gamma)], [T3 (temporal), fam 8∼16 Hz (alpha), and fc 8∼16 Hz (alpha)], [P4 (parietal) *f*am 4∼8 Hz (theta), and *f*c 4∼8 Hz (theta)]. Three features were inconsistent with the AM-FM power map, including the second (F4 channel), third (F4 channel), and sixth (P4 channel). However, these features are adjacent to the color squares, in which some matched features may also have been omitted during the decorrelation process in the feature selection stage (Agarwal, 2019).

When comparing MCI vs. CN, carrier frequencies belonging to the lower frequency-modulating HFO (i.e., two beta and one gamma) are more important than the LFO (i.e., delta2 and theta). In AD1 vs. CN, carrier frequencies in the lower frequency-modulating HFO (alpha, beta, and two gammas) are more essential than the LFO (delta1 and theta; Table 5). In the longitudinal MCI cohort dataset, the validated features and classifiers can be used to screen probable converted MCI with SVM, followed by Decision Tree to boost relatively low accuracy in the prediction of MCI conversion (Hampel et al., 2011).

## 5. The potential neurobiological mechanisms of altered brain oscillations in neurodegenerative disease

The pattern of altered brain oscillations in AD patients, particularly the augmented LFO AM power coupled with attenuated HFO AM power, is reminiscent of EEG slowing in patients with dementia of Lewy bodies (DLB; van der Zande et al., 2018, 2020) and a common pattern of EEG signatures in patients with major vascular cognitive impairment (VCI; Babiloni et al., 2021). These include slowing of peak frequency and increased LFO with decreased relative alpha activity (d’Onofrio et al., 1996), decreased power of posterior beta activity with decreased frontal HFO power (Holschneider and Leuchter, 1995), and increased widespread LFO power (Neto et al., 2015). EEG slowing in DLB is associated with severe cholinergic neuron degeneration (van der Zande et al., 2020), whereas the resemblance of altered brain oscillations between AD and major VCI insinuates the presence of an overlapping vulnerable region contributing to brain oscillation changes due to basal forebrain dysfunction or cholinergic depletion (Román and Kalaria, 2006; Wang et al., 2009). This is seen not only in selectively vulnerable disease-specific neurons in AD but also in PD with dementia, DLB (Pepeu et al., 2015; Ballinger et al., 2016; Fu et al., 2018), or ischemic long-tract projections of the basal forebrain in subcortical small vessel disease (Engelhardt et al., 2007; Pepeu et al., 2015; Ballinger et al., 2016). Thus, lower-frequencies power augmentation is hypothesized to reflect the ensemble representation of interplay amid cholinergic deficit and reduced rCBF based on evidence of the interaction of Aβ, basal forebrain, and neurovascular uncoupling in AD (Schliebs and Arendt, 2011: Pepeu et al., 2015: Love and Miners, 2016; Iadecola, 2017) and mixed pathology in AD (Schneider et al., 2007; Schreiter Gasser et al., 2008; Arvanitakis et al., 2016). The attenuated HFO (alpha and beta) are highly correlated with the underlying neuropathological change of tauopathy in AD and frontotemporal dementia (Smailovic et al., 2018), which share a similar topographical distribution of neurofibrillary tangles and comparable decreased alpha and beta power in rsEEG (Lindau et al., 2003; Nishida et al., 2011). Our results here show that these signatures could be predictive markers for disease progression.

When considering the gamma band, the time-varying power fluctuation of the fc (band-limited power; BLP) likely signifies potential functional connectivity of spatial specificity in the cerebral cortex (Leopold et al., 2003). Possible aberrant neuromodulation between AM and pre-specified fc bandwidths (beta and theta bands) could differentiate mild AD patients from CN (Fraga et al., 2013). They both computed rsEEG signals analogously to our method, providing the AM or second spectrum of given frequency bands and delineating the interdependency between the primary and second spectrum (Leopold et al., 2003; Falk et al., 2012; Huang et al., 2016). Electrocorticogram (ECoG) recording conducted in preoperative epileptic patients validates that slow BLP of gamma oscillations exhibit a high correlation between mirrored sites of both hemispheres (Nir et al., 2008), suggesting interhemispheric functional connectivity (Smith et al., 2009). This ushers a mechanistic question (Drew et al., 2008): What is the very slow frequency modulatory source of gamma oscillations (Drew et al., 2020)? Mateo et al. (2017) modeled this problem in mice with concurrent two-photon imaging of arteriole diameter and LFP measurements with an optogenetic approach, elucidating that gamma-band oscillations modulated arteriole vasomotion at a very slow frequency (0.1 Hz), contributing to changes in BOLD signals of resting-state fMRI (Mateo et al., 2017). Furthermore, a lesion study in monkeys showed that the basal forebrain mediates global spontaneous resting-state fMRI fluctuations (Turchi et al., 2018) and findings of anticholinergic agent-provocative EEG index negatively correlated with the severity of neurodegenerative diseases (Johannsson et al., 2015). These pieces of evidence suggest that the cholinergic system plays an essential role in the amplitude modulation of brain oscillations and vasomotion control (Lecrux et al., 2017; Drew et al., 2020).

Based on the cholinergic hypothesis in the fundamentals of developing neuropsychiatric symptoms of AD, cholinesterase inhibitors can not only improve cognitive function (Hampel et al., 2018) but also reverse and lessen altered brain oscillations in AD patients (Brassen and Adler, 2003; Rodriguez et al., 2004; Babiloni et al., 2006a; Gianotti et al., 2008). The augmented posterior alpha oscillations found in our study and others (Huang et al., 2000; Luckhaus et al., 2008) in MCI-S might represent a compensatory mechanism for early cognitive decline with enhanced cholinergic activity as the most likely neurobiological basis (Dekosky et al., 2002).

## 6. Limitations

A somewhat sparse electrode array (i.e., 19 electrodes) was used for the recording of the rsEEG. This could limit conclusions about scalp distributions and topographical descriptions in the altered power patterns in the AM-FM energy map. The Nihon Kohden amplifier with 19 channels is commonly used in the clinical setting of hospitals, which is the source of our participants’ dataset and is definitely a limitation in depicting the topographical pattern of rsEEG activity (Wang et al., 2023). Therefore, we registered the significant power density in the AF-FM map with channel locations but concluded the important findings in the brain regions.

This study also used a retrospective cross-sectional design, meaning that only available data at hand could be incorporated into the analyses. For future work, it would be good to have records that include biological fluid collection (e.g., blood and CSF), medication consumption, neuropsychiatric scales (e.g., neuropsychiatric inventory, Beck Depression Inventory, Hamilton depression score, etc.), and other neuroimaging modalities (e.g., FDG-PET, MRI, and MEG), if possible, for further correlation/regression analyses.

## 7. Conclusion and future work

Attenuated AM power of alpha and beta oscillations at MCI can predict the future progression to AD. Attenuated beta and gamma AM power densities with augmented delta and theta AM power densities are prominent in early cognitive decline and can serve as target oscillations for therapeutic intervention. The HHSA-based feature extraction outperformed other analytical methods when deploying machine learning algorithms. Therefore, integrating HHSA of EEG signals into machine learning algorithms can be a clinical tool to differentiate between CN individuals and patients with cognitive decline and predict the conversion from MCI to AD. As the HHSA can wholly and efficiently depict these potential interactions, integrating the evidence of neurobiological profiles across the AD spectrum together with a correlation of brain oscillatory signatures could provide a non-invasive, affordable, and reproducible surrogate marker for screening, detecting, and follow-up in large populations with early cognitive decline.

## Data availability statement

The original contributions presented in this study are included in the article/Supplementary material, further inquiries can be directed to the corresponding author.

## Ethics statement

The studies involving humans were approved by the Chi-Mei Medical Center Institutional Review Board, Taipei VGH Institutional Review Board. The studies were conducted in accordance with the local legislation and institutional requirements. Written informed consent for participation in this study was provided by the participants’ legal guardians/next of kin.

## Author contributions

K-TC and C-HJ contributed to the conception, organization, resources, and finalization of the manuscript. C-FC, W-CL, W-SC, W-KL, NH, and C-HJ were performed the EEG and their analysis. K-TC, M-HW, J-LF, and S-JW recruited the patients and examined the patients and controls. K-TC, C-FC, W-CL, IF, C-HJ, W-KL, and NH performed the statistical analysis. C-HJ acquired the funding and supervised the manuscript. K-TC and IF wrote the first draft. All authors reviewed and critiqued the manuscript.

## References

[B1] Adaikkan C. Tsai L.-H. (2020). Gamma entrainment: Impact on neurocircuits, glia, and therapeutic opportunities. *Trends Neurosci.* 43 24–41. 10.1016/j.tins.2019.11.001 31836315

[B2] Agarwal R. (2019). *The 5 feature selection algorithms every data scientist should know*. ML Wiz. Available online at: https://towardsdatascience.com/the-5-feature-selection-algorithms-every-data-scientist-need-to-know-3a6b566efd2 (accessed July 27, 2023).

[B3] Arvanitakis Z. Capuano A. W. Leurgans S. E. Bennett D. A. Schneider J. A. (2016). Relation of cerebral vessel disease to Alzheimer’s disease dementia and cognitive function in elderly people: A cross-sectional study. *Lancet Neurol.* 15 934–943. 10.1016/S1474-4422(16)30029-1 27312738 PMC4969105

[B4] Babiloni C. Arakaki X. Bonanni L. Bujan A. Carrillo M. C. Del Percio C. (2021). EEG measures for clinical research in major vascular cognitive impairment: Recommendations by an expert panel. *Neurobiol. Aging* 103 78–97. 10.1016/j.neurobiolaging.2021.03.003 33845399

[B5] Babiloni C. Cassetta E. Binetti G. Tombini M. Del Percio C. Ferreri F. (2007). Resting EEG sources correlate with attentional span in mild cognitive impairment and Alzheimer’s disease. *Eur. J. Neurosci.* 25 3742–3757. 10.1111/j.1460-9568.2007.05601.x 17610594

[B6] Babiloni C. Cassetta E. Dal Forno G. Del Percio C. Ferreri F. Ferri R. (2006a). Donepezil effects on sources of cortical rhythms in mild Alzheimer’s disease: Responders vs. non-responders. *Neuroimage* 31 1650–1665. 10.1016/j.neuroimage.2006.02.015 16600641

[B7] Babiloni C. Binetti G. Cassetta E. Dal Forno G. Del Percio C. Ferreri F. (2006b). Sources of cortical rhythms change as a function of cognitive impairment in pathological aging: A multicenter study. *Clin. Neurophysiol.* 117 252–268. 10.1016/j.clinph.2005.09.01 16377238

[B8] Babiloni C. Ferri R. Binetti G. Cassarino A. Forno G. D. Ercolani M. (2006c). Fronto-parietal coupling of brain rhythms in mild cognitive impairment: A multicentric EEG study. *Brain Res. Bull.* 69 63–73. 10.1016/j.brainresbull.2005.10.013 16464686

[B9] Babiloni C. Benussi L. Binetti G. Cassetta E. Dal Forno G. Del Percio C. (2006d). Apolipoprotein E and alpha brain rhythms in mild cognitive impairment: A multicentric electroencephalogram study. *Ann. Neurol.* 59 323–334. 10.1002/ana.20724 16358334

[B10] Babiloni C. Binetti G. Cassarino A. Dal Forno G. Del Percio C. Ferreri F. (2006e). Sources of cortical rhythms in adults during physiological aging: A multicentric EEG study. *Hum. Brain Mapp.* 27 162–172. 10.1002/hbm.20175 16108018 PMC6871339

[B11] Babiloni C. De Pandis M. F. Vecchio F. Buffo P. Sorpresi F. Frisoni G. B. (2011). Cortical sources of resting state electroencephalographic rhythms in Parkinson’s disease related dementia and Alzheimer’s disease. *Clin. Neurophysiol.* 122 2355–2364. 10.1016/j.clinph.2011.03.029 21924950

[B12] Babiloni C. Del Percio C. Bordet R. Bourriez J. L. Bentivoglio M. Payoux P. (2013). Effects of acetylcholinesterase inhibitors and memantine on resting-state electroencephalographic rhythms in Alzheimer’s disease patients. *Clin. Neurophysiol.* 124 837–850. 10.1016/j.clinph.2012.09.017 23098644

[B13] Babiloni C. Del Percio C. Caroli A. Salvatore E. Nicolai E. Marzano N. (2016). Cortical sources of resting state EEG rhythms are related to brain hypometabolism in subjects with Alzheimer’s disease: An EEG-PET study. *Neurobiol. Aging* 48 122–134. 10.1016/j.neurobiolaging.2016.08.021 27668356

[B14] Babiloni C. Del Percio C. Lizio R. Noce G. Cordone S. Lopez S. (2017). Abnormalities of cortical neural synchronization mechanisms in patients with dementia due to Alzheimer’s and Lewy body diseases: An EEG study. *Neurobiol. Aging* 55 143–158. 10.1016/j.neurobiolaging.2017.03.030 28454845

[B15] Babiloni C. Del Percio C. Lizio R. Noce G. Lopez S. Soricelli A. (2018). Abnormalities of resting-state functional cortical connectivity in patients with dementia due to Alzheimer’s and Lewy body diseases: An EEG study. *Neurobiol. Aging* 65 18–40. 10.1016/j.neurobiolaging.2017.12.023 29407464

[B16] Ballinger E. C. Ananth M. Talmage D. A. Role L. W. (2016). Basal forebrain cholinergic circuits and signaling in cognition and cognitive decline. *Neuron* 91 1199–1218. 10.1016/j.neuron.2016.09.006 27657448 PMC5036520

[B17] Bateman R. J. Xiong C. Benzinger T. L. S. Fagan A. M. Goate A. Fox N. C. (2012). Clinical and biomarker changes in dominantly inherited Alzheimer’s disease. *N. Engl. J. Med.* 367 795–804. 10.1056/nejmoa1202753 22784036 PMC3474597

[B18] Bedrosian E. (1963). “A product theorem for Hilbert transforms,” in *Proceedings of the IEEE*, (Piscataway, NJ: IEEE), 868–869.

[B19] Brassen S. Adler G. (2003). Short-term effects of acetylcholinesterase inhibitor treatment on EEG and memory performance in Alzheimer patients: An open, controlled trial. *Pharmacopsychiatry* 36 304–308. 10.1055/s-2003-45118 14663655

[B20] Canolty R. T. Knight R. T. (2010). The functional role of cross-frequency coupling. *Trends Cogn. Sci*. 14, 506–515. 20932795 10.1016/j.tics.2010.09.001 PMC3359652

[B21] Caravaglios G. Muscoso E. G. Di Maria G. Costanzo E. (2015). Patients with mild cognitive impairment have an abnormal upper-alpha event-related desynchronization/synchronization (ERD/ERS) during a task of temporal attention. *J. Neural Transm.* 122 441–453. 10.1007/s00702-014-1262-7 24947877

[B22] Cassani R. Falk T. H. (2019). Spectrotemporal modeling of biomedical signals: Theoretical foundation and applications. *Encyclopedia Biomed. Eng.* 1–3 144–163. 10.1016/B978-0-12-801238-3.99993-8

[B23] Colominas M. A. Schlotthauer G. Torres M. E. (2014). Improved complete ensemble EMD: A suitable tool for biomedical signal processing. *Biomed. Signal Process. Control* 14 19–29. 10.1016/j.bspc.2014.06.009

[B24] Colominas M. A. Schlotthauer G. Torres M. E. Flandrin P. (2012). Noise-assisted EMD methods in action. *Adv. Adaptive Data Anal.* 4:1250025. 10.1142/s1793536912500252

[B25] Cook I. A. Leuchter A. F. (1996). Synaptic dysfunction in Alzheimer’s disease: Clinical assessment using quantitative EEG. *Behav. Brain Res.* 78 15–23. 8793033 10.1016/0166-4328(95)00214-6

[B26] d’Onofrio F. Salvia S. Petretta V. Bonavita V. Rodriguez G. Tedeschi G. (1996). Quantified-EEG in normal aging and dementias. *Acta Neurol. Scand.* 93 336–345. 10.1111/j.1600-0404.1996.tb00006.x 8800344

[B27] Dauwels J. Vialatte F. Cichocki A. (2010). Diagnosis of Alzheimer’s disease from EEG signals: Where are we standing? *Curr. Alzheimer Res.* 7 487–505. 20455865 10.2174/156720510792231720

[B28] Dekosky S. T. Ikonomovic M. D. Styren S. D. Beckett L. Wisniewski S. Bennett D. A. (2002). Upregulation of choline acetyltransferase activity in hippocampus and frontal cortex of elderly subjects with mild cognitive impairment. *Ann. Neurol.* 51 145–155. 10.1002/ana.10069 11835370

[B29] Donoghue T. Haller M. Peterson E. J. Varma P. Sebastian P. Gao R. (2020). Parameterizing neural power spectra into periodic and aperiodic components. *Nat. Neurosci.* 23 1655–1665. 33230329 10.1038/s41593-020-00744-x PMC8106550

[B30] Drew P. J. Duyn J. H. Golanov E. Kleinfeld D. (2008). Finding coherence in spontaneous oscillations. *Nat. Neurosci.* 11 991–993. 10.1038/nn0908-991 18725901

[B31] Drew P. J. Mateo C. Turner K. L. Yu X. Kleinfeld D. (2020). Ultra-slow oscillations in fMRI and resting-state connectivity: Neuronal and vascular contributions and technical confounds. *Neuron* 107 782–804. 10.1016/j.neuron.2020.07.020 32791040 PMC7886622

[B32] Dubois B. Epelbaum S. Nyasse F. Bakardjian H. Gagliardi G. Uspenskaya O. (2018). Cognitive and neuroimaging features and brain β-amyloidosis in individuals at risk of Alzheimer’s disease (INSIGHT-preAD): A longitudinal observational study. *Lancet Neurol.* 17 335–346. 10.1016/S1474-4422(18)30029-2 29500152

[B33] Engel A. K. Fries P. König P. Brecht M. Singer W. (1999). Temporal binding, binocular rivalry, and consciousness. *Conscious. Cogn.* 8 128–51. 10447995 10.1006/ccog.1999.0389

[B34] Engelhardt E. Moreira D. M. Laks J. (2007). Vascular dementia and the cholinergic pathways. *Dement. Neuropsychol.* 1 2–9. 10.1590/S1980-57642008DN10100002 29213361 PMC5619377

[B35] Falk T. H. Fraga F. J. Trambaiolli L. Anghinah R. (2012). EEG amplitude modulation analysis for semi-automated diagnosis of Alzheimer’s disease. *Eurasip J. Adv. Signal Process.* 2012:192. 10.1186/1687-6180-2012-192

[B36] Fiebelkorn I. C. Kastner S. (2018). A rhythmic theory of attention. *Trends Cogn. Sci.* 23 87–101. 30591373 10.1016/j.tics.2018.11.009 PMC6343831

[B37] Flandrin P. Rilling G. Goncalves P. (2004). Empirical mode decomposition as a filter bank. *IEEE Signal Process. Lett.* 11 112–114.

[B38] Folstein M. F. Folstein S. E. McHugh P. R. (1975). “Mini-mental state”. A practical method for grading the cognitive state of patients for the clinician. *J. Psychiatric Res.* 12 189–198. 10.1016/0022-3956(75)90026-6 1202204

[B39] Fraga F. J. Falk T. H. Kanda P. A. M. Anghinah R. (2013). Characterizing Alzheimer’s disease severity via resting-awake EEG amplitude modulation analysis. *PLoS One* 8:e72240. 10.1371/journal.pone.0072240 24015222 PMC3754998

[B40] Fries P. Reynolds J. H. Rorie A. E. Desimone R. (2001). Modulation of oscillatory neuronal synchronization by selective visual attention. *Science* 291 1560–1563. 10.1126/science.1055465 11222864

[B41] Friese U. Köster M. Hassler U. Martens U. Trujillo-Barreto N. Gruber T. (2013). Successful memory encoding is associated with increased cross-frequency coupling between frontal theta and posterior gamma oscillations in human scalp-recorded EEG. *Neuroimage* 66 642–647. 23142278 10.1016/j.neuroimage.2012.11.002

[B42] Fu H. Hardy J. Duff K. E. (2018). Selective vulnerability in neurodegenerative diseases. *Nat. Neurosci.* 21 1350–1358. 10.1038/s41593-018-0221-2 30250262 PMC6360529

[B43] Gao R. Peterson E. J. Voytek B. (2017). Inferring synaptic excitation/inhibition balance from field potentials. *Neuroimage* 158 70–78. 28676297 10.1016/j.neuroimage.2017.06.078

[B44] Gianotti L. R. R. Künig G. Faber P. L. Lehmann D. Pascual-Marqui R. D. Kochi K. (2008). Rivastigmine effects on EEG spectra and three-dimensional LORETA functional imaging in Alzheimer’s disease. *Psychopharmacology* 198 323–332. 10.1007/s00213-008-1111-1 18446328

[B45] Gilberet R. C. M. P. Roy R. S. Sairamya N. J. Ponraj D. N. George S. T. (2017). “Automated artifact rejection using ICA and image processing algorithms,” in *2017 International conference on signal processing and communication (ICSPC)*, (Piscataway, NJ: IEEE), 354–358. 10.1109/CSPC.2017.8305868

[B46] Groppe D. M. Urbach T. P. Kutas M. (2011). Mass univariate analysis of event-related brain potentials/fields I: A critical tutorial review. *Psychophysiology* 48 1711–1725. 10.1111/j.1469-8986.2011.01273.x 21895683 PMC4060794

[B47] Grover S. Nguyen J. A. Reinhart R. M. G. (2021). Synchronizing brain rhythms to improve cognition. *Annu. Rev. Med.* 72 29–43. 10.1146/annurev-med-060619-022857 33035432 PMC10068593

[B48] Guyon I. Elisseeff A. (2003). An introduction to variable and feature selection. *J. Mach. Learn. Res.* 3 1157–1182.

[B49] Hampel H. Mesulam M. M. Cuello A. C. Farlow M. R. Giacobini E. Grossberg G. T. (2018). The cholinergic system in the pathophysiology and treatment of Alzheimer’s disease. *Brain* 141 1917–1933. 10.1093/brain/awy132 29850777 PMC6022632

[B50] Hampel H. Prvulovic D. Teipel S. Jessen F. Luckhaus C. Frölich L. (2011). The future of Alzheimer’s disease: The next 10 years. *Prog. Neurobiol.* 95 718–728. 10.1016/j.pneurobio.2011.11.008 22137045

[B51] He B. J. (2014). Scale-free brain activity: Past, present, and future. *Trends Cogn. Sci.* 18 480–487. 24788139 10.1016/j.tics.2014.04.003 PMC4149861

[B52] Herholz K. (2010). Cerebral glucose metabolism in preclinical and prodromal Alzheimers disease. *Expert Rev. Neurother.* 10 1667–1673. 10.1586/ern.10.136 20977325

[B53] Holschneider D. P. Leuchter A. F. (1995). Beta activity in aging and dementia. *Brain Topogr.* 8 169–180. 8793127 10.1007/BF01199780

[B54] Hsiao F. J. Wang Y. J. Yan S. H. Chen W. T. Lin Y. Y. (2013). Altered oscillation and synchronization of default-mode network activity in mild Alzheimer’s disease compared to mild cognitive impairment: An electrophysiological study. *PLoS One* 8:e68792. 10.1371/journal.pone.0068792 23874766 PMC3708894

[B55] Huang C. Wahlund L.-O. Dierks T. Julin P. Winblad B. Jelic V. (2000). Discrimination of Alzheimer’s disease and mild cognitive impairment by equivalent EEG sources: A cross-sectional and longitudinal study. *Clin. Neurophysiol.* 111 1961–1967. 10.1016/S1388-2457(00)00454-5 11068230

[B56] Huang N. E. Hu K. Yang A. C. C. Chang H. C. Jia D. Liang W. K. (2016). On Holo-Hilbert spectral analysis: A full informational spectral representation for nonlinear and non-stationary data. *Philos. Trans. R. Soc. A Math. Phys. Eng. Sci.* 374:20150206. 10.1098/rsta.2015.0206 26953180 PMC4792412

[B57] Huang N. E. Shen Z. Long S. R. Wu M. C. Shih H. H. Zheng Q. (1998). The empirical mode decomposition and the Hilbert spectrum for nonlinear and non-stationary time series analysis. *Proc. R. Soc. Lond. Series A Math. Phys. Eng. Sci.* 454 903–995. 10.1098/rspa.1998.0193 26953177

[B58] Huang N. E. Wu Z. Long S. R. Arnold K. C. Chen X. Blank K. (2009). On instantaneous frequency. *Adv. Adapt Data Anal*. 1, 177–229.

[B59] Iaccarino H. F. Singer A. C. Martorell A. J. Rudenko A. Gao F. Gillingham T. Z. (2016). Gamma frequency entrainment attenuates amyloid load and modifies microglia. *Nature* 540 230–235. 10.1038/nature20587 27929004 PMC5656389

[B60] Iadecola C. (2017). The neurovascular unit coming of age: A journey through neurovascular coupling in health and disease. *Neuron* 96 17–42. 10.1016/j.neuron.2017.07.030 28957666 PMC5657612

[B61] Jack C. R. Bennett D. A. Blennow K. Carrillo M. C. Dunn B. Haeberlein S. B. (2018). NIA-AA research framework: Toward a biological definition of Alzheimer’s disease. *Alzheimers Dement.* 14 535–562. 10.1016/j.jalz.2018.02.018 29653606 PMC5958625

[B62] Jelic V. Shigeta M. Julin P. Almkvist O. Winblad B. Wahlund L.-O. (1996). Quantitative electroencephalography power and coherence in Alzheimer’s disease and mild cognitive impairment. *Dement. Geriatr. Cogn. Disord.* 7 314–323. 10.1159/000106897 8915037

[B63] Johannsson M. Snaedal J. Johannesson G. H. Gudmundsson T. E. Johnsen K. (2015). The acetylcholine index: An electroencephalographic marker of cholinergic activity in the living human brain applied to Alzheimer’s disease and other dementias. *Dement. Geriatr. Cogn. Disord.* 39 132–142. 10.1159/000367889 25471612

[B64] Juan C. H. Nguyen K. T. Liang W. K. Quinn A. J. Chen Y. H. Muggleton N. G. (2021). Revealing the dynamic nature of amplitude modulated neural entrainment with Holo-Hilbert spectral analysis. *Front. Neurosci.* 15:673369. 10.3389/fnins.2021.673369 34421511 PMC8375503

[B65] Klimesch W. (2018). The frequency architecture of brain and brain body oscillations: An analysis. *Eur. J. Neurosci.* 48 2431–2453. 30281858 10.1111/ejn.14192 PMC6668003

[B66] Klimesch W. Sauseng P. Hanslmayr S. (2007). EEG alpha oscillations: The inhibition-timing hypothesis. *Brain Res. Rev.* 53 63–88. 10.1016/j.brainresrev.2006.06.003 16887192

[B67] Lakatos P. Shah A. S. Knuth K. H. Ulbert I. Karmos G. Schroeder C. E. (2005). An oscillatory hierarchy controlling neuronal excitability and stimulus processing in the auditory cortex. *J. Neurophysiol*. 94, 1904–1911. 15901760 10.1152/jn.00263.2005

[B68] Lecrux C. Sandoe C. H. Neupane S. Kropf P. Toussay X. Tong X. K. (2017). Impact of altered cholinergic tones on the neurovascular coupling response to whisker stimulation. *J. Neurosci.* 37 1518–1531. 10.1523/JNEUROSCI.1784-16.2016 28069927 PMC6705676

[B69] Lehmann D. Skrandies W. (1980). Reference-free identification of components of checkerboard-evoked multichannel potential fields. *Electroencephalogr. Clin. Neurophysiol.* 48 609–621. 10.1016/0013-4694(80)90419-8 6155251

[B70] Leopold D. A. Murayama Y. Logothetis N. K. (2003). Very slow activity fluctuations in monkey visual cortex: Implications for functional brain imaging. *Cereb. Cortex* 13 422–433. 10.1093/CERCOR/13.4.422 12631571

[B71] Liang W. K. Tseng P. Yeh J. R. Huang N. E. Juan C. H. (2021). Frontoparietal beta amplitude modulation and its interareal cross-frequency coupling in visual working memory. *Neuroscience* 460 69–87. 10.1016/j.neuroscience.2021.02.013 33588001

[B72] Lin K. N. Liu H. C. (2003). Clinical dementia rating (CDR), Chinese version. *Acta Neurol. Taiwanica* 12 154–165.

[B73] Lindau M. Jelic V. Johansson S. E. Andersen C. Wahlund L. O. Almkvist O. (2003). Quantitative EEG abnormalities and cognitive dysfunctions in frontotemporal dementia and Alzheimer’s disease. *Dement Geriatr. Cogn. Disord.* 15 106–114. 10.1159/000067973 12566600

[B74] Linkenkaer-Hansen K. Nikouline V. V. Matias Palva J. Ilmoniemi R. J. (2001). Long-range temporal correlations and scaling behavior in human brain oscillations. *J. Neurosci.* 21 1370–1377. 11160408 10.1523/JNEUROSCI.21-04-01370.2001 PMC6762238

[B75] Llinás R. Ribary U. (1993). Coherent 40-Hz oscillation characterizes dream state in humans. *Proc. Natl. Acad. Sci. U.S.A.* 90 2078–2081. 10.1073/pnas.90.5.2078 8446632 PMC46024

[B76] Love S. Miners J. S. (2016). Cerebrovascular disease in ageing and Alzheimer’s disease. *Acta Neuropathol.* 131 645–658. 10.1007/s00401-015-1522-0 26711459 PMC4835514

[B77] Luckhaus C. Grass-Kapanke B. Blaeser I. Ihl R. Supprian T. Winterer G. (2008). Quantitative EEG in progressing vs stable mild cognitive impairment (MCI): Results of a 1-year follow-up study. *Int. J. Geriatr. Psychiatry* 23 1148–1155. 10.1002/gps.2042 18537220

[B78] Maestú F. Cuesta P. Hasan O. Fernandéz A. Funke M. Schulz P. E. (2019). The importance of the validation of M/EEG with current biomarkers in Alzheimer’s disease. *Front. Hum. Neurosci.* 13:17. 10.3389/fnhum.2019.00017 30792632 PMC6374629

[B79] Maris E. Oostenveld R. (2007). Nonparametric statistical testing of EEG- and MEG-data. *J. Neurosci. Methods* 164 177–190. 10.1016/j.jneumeth.2007.03.024 17517438

[B80] Maris E. Schoffelen J. M. Fries P. (2007). Nonparametric statistical testing of coherence differences. *J. Neurosci. Methods* 163 161–175. 10.1016/j.jneumeth.2007.02.011 17395267

[B81] Mateo C. Knutsen P. M. Tsai P. S. Shih A. Y. Kleinfeld D. (2017). Entrainment of arteriole vasomotor fluctuations by neural activity is a basis of blood-oxygenation-level-dependent “Resting-State” connectivity. *Neuron* 96 936–948.e3. 10.1016/j.neuron.2017.10.012 29107517 PMC5851777

[B82] McDade E. Bateman R. J. (2017). Stop Alzheimer’s before it starts. *Nature* 547 153–155. 10.1038/547153a 28703214

[B83] McKhann G. Drachman D. Folstein M. Katzman R. Price D. Stadlan E. M. (1984). Clinical diagnosis of Alzheimer’s disease: Report of the NINCDS-ADRDA work group under the auspices of department of health and human services task force on Alzheimer’s disease. *Neurology* 34 939–944. 10.1212/WNL.34.7.939 6610841

[B84] Merkin A. Sghirripa S. Graetz L. Smith A. E. Hordacre B. Harris R. (2023). Do age-related differences in aperiodic neural activity explain differences in resting EEG alpha? *Neurobiol. Aging* 121 78–87. 36379095 10.1016/j.neurobiolaging.2022.09.003

[B85] Moretti D. V. Pievani M. Fracassi C. Binetti G. Rosini S. Geroldi C. (2009). Increase of theta/gamma and alpha3/alpha2 ratio is associated with amygdalo-hippocampal complex atrophy. *J. Alzheimers Dis.* 17 349–357. 19363263 10.3233/JAD-2009-1059

[B86] Moretti D. V. Pievani M. Pini L. Guerra U. P. Paghera B. Frisoni G. B. (2017). Cerebral PET glucose hypometabolism in subjects with mild cognitive impairment and higher EEG high-alpha/low-alpha frequency power ratio. *Neurobiol. Aging* 58 213–224. 10.1016/j.neurobiolaging.2017.06.009 28755648

[B87] Moruzzi G. Magoun H. W. (1949). Brain stem reticular formation and activation of the EEG. *Electroencephalogr. clin. neurophysiol*. 1, 455–473. 18421835

[B88] Munk M. H. J. Roelfsema P. R. König P. Engel A. K. Singer W. (1996). Role of reticular activation in the modulation of intracortical synchronization. *Science* 272 271–274. 10.1126/science.272.5259.271 8602512

[B89] Musaeus C. S. Engedal K. Høgh P. Jelic V. Mørup M. Naik M. (2018a). EEG theta power is an early marker of cognitive decline in dementia due to Alzheimer’s disease. *J. Alzheimers Dis.* 64 1359–1371. 10.3233/JAD-180300 29991135

[B90] Musaeus C. S. Nielsen M. S. Østerbye N. N. Høgh P. (2018b). Decreased parietal beta power as a sign of disease progression in patients with mild cognitive impairment. *J. Alzheimers Dis.* 65 475–487. 10.3233/JAD-180384 30056426

[B91] Nakamura A. Cuesta P. Fernández A. Arahata Y. Iwata K. Kuratsubo I. (2018). Electromagnetic signatures of the preclinical and prodromal stages of Alzheimer’s disease. *Brain* 141 1470–1485. 10.1093/brain/awy044 29522156 PMC5920328

[B92] Neto E. Allen E. A. Aurlien H. Nordby H. Eichele T. (2015). EEG spectral features discriminate between Alzheimer’s and vascular dementia. *Front. Neurol.* 6:25. 10.3389/fneur.2015.00025 25762978 PMC4327579

[B93] Nguyen K. T. Liang W. K. Lee V. Chang W. S. Muggleton N. G. Yeh J. R. (2019). Unraveling nonlinear electrophysiologic processes in the human visual system with full dimension spectral analysis. *Sci. Rep.* 9:16919. 10.1038/s41598-019-53286-z 31729410 PMC6858326

[B94] Nir Y. Mukamel R. Dinstein I. Privman E. Harel M. Fisch L. (2008). Interhemispheric correlations of slow spontaneous neuronal fluctuations revealed in human sensory cortex. *Nat. Neurosci.* 11 1100–1108. 10.1038/nn.2177 19160509 PMC2642673

[B95] Nishida K. Yoshimura M. Isotani T. Yoshida T. Kitaura Y. Saito A. (2011). Differences in quantitative EEG between frontotemporal dementia and Alzheimer’s disease as revealed by LORETA. *Clin. Neurophysiol.* 122 1718–1725. 10.1016/j.clinph.2011.02.011 21396882

[B96] Osipova D. Hermes D. Jensen O. (2008). Gamma power is phase-locked to posterior alpha activity. *PLoS One* 3:e3990. 10.1371/journal.pone.0003990 19098986 PMC2602598

[B97] Palop J. J. Mucke L. (2016). Network abnormalities and interneuron dysfunction in Alzheimer disease. *Nat. Rev. Neurosci*. 17, 777–792. 27829687 10.1038/nrn.2016.141 PMC8162106

[B98] Pepeu G. Grossi C. Casamenti F. (2015). The brain cholinergic system in neurodegenerative diseases. *Annu. Res. Rev. Biol.* 6 1–19. 10.9734/arrb/2015/14623

[B99] Petrova T. Orellana C. Jelic V. Oeksengaard A. R. Snaedal J. Høgh P. (2020). Cholinergic dysfunction, neurodegeneration, and amyloid-beta pathology in neurodegenerative diseases. *Psychiatry Res. Neuroimaging* 302:111099. 10.1016/j.pscychresns.2020.111099 32505903

[B100] Poil S. S. de Haan W. van der Flier W. M. Mansvelder H. D. Scheltens P. Linkenkaer-Hansen K. (2013). Integrative EEG biomarkers predict progression to Alzheimer’s disease at the MCI stage. *Front. Aging Neurosci.* 5:58. 10.3389/fnagi.2013.00058 24106478 PMC3789214

[B101] Quinn A. J. Lopes-Dos-Santos V. Dupret D. Nobre A. C. (2021). EMD: Empirical mode decomposition and Hilbert-Huang spectral analyses in python. *J. Open Source Softw*. 6:2977. 10.21105/joss.02977 PMC7610596 33855259

[B102] Reinhart R. M. G. Nguyen J. A. (2019). Working memory revived in older adults by synchronizing rhythmic brain circuits. *Nat. Neurosci.* 22 820–827. 10.1038/s41593-019-0371-x 30962628 PMC6486414

[B103] Rodriguez G. Vitali P. Canfora M. Calvini P. Girtler N. De Leo C. (2004). Quantitative EEG and perfusional single photon emission computed tomography correlation during long-term donepezil therapy in Alzheimer’s disease. *Clin. Neurophysiol.* 115 39–49. 10.1016/S1388-2457(03)00321-3 14706467

[B104] Román G. C. Kalaria R. N. (2006). Vascular determinants of cholinergic deficits in Alzheimer disease and vascular dementia. *Neurobiol. Aging* 27 1769–1785. 10.1016/j.neurobiolaging.2005.10.004 16300856

[B105] Schliebs R. Arendt T. (2011). The cholinergic system in aging and neuronal degeneration. *Behav. Brain Res.* 221 555–563. 10.1016/j.bbr.2010.11.058 21145918

[B106] Schneider J. A. Boyle P. A. Arvanitakis Z. Bienias J. L. Bennett D. A. (2007). Subcortical infarcts, Alzheimer’s disease pathology, and memory function in older persons. *Ann. Neurol.* 62 59–66. 10.1002/ana.21142 17503514

[B107] Schreiter Gasser U. Rousson V. Hentschel F. Sattel H. Gasser T. (2008). Alzheimer disease versus mixed dementias: An EEG perspective. *Clin. Neurophysiol.* 119 2255–2259. 10.1016/j.clinph.2008.07.216 18768349

[B108] Segal D. L. (2010). “Diagnostic and statistical manual of mental disorders (DSM-IV-TR),” in *The Corsini encyclopedia of psychology*, eds Chu-Lien Chao R. Manita J. (Atlanta, GA: American Cancer Society). 10.1002/9780470479216.CORPSY0271

[B109] Smailovic U. Jelic V. (2019). Neurophysiological markers of Alzheimer’s disease: Quantitative EEG approach. *Neurol. Ther.* 8 37–55. 10.1007/s40120-019-00169-0 31833023 PMC6908537

[B110] Smailovic U. Koenig T. Kåreholt I. Andersson T. Kramberger M. G. Winblad B. (2018). Quantitative EEG power and synchronization correlate with Alzheimer’s disease CSF biomarkers. *Neurobiol. Aging* 63 88–95. 10.1016/j.neurobiolaging.2017.11.005 29245058

[B111] Smith S. M. Fox P. T. Miller K. L. Glahn D. C. Fox P. M. Mackay C. E. (2009). Correspondence of the brain’s functional architecture during activation and rest. *Proc. Natl. Acad. Sci. U.S.A.* 106 13040–13045. 10.1073/pnas.0905267106 19620724 PMC2722273

[B112] Steriade M. Timofeev I. (2003). Neuronal plasticity in thalamocortical networks during sleep and waking oscillations. *Neuron* 37 563–576. 12597855 10.1016/s0896-6273(03)00065-5

[B113] Tallon-Baudry C. Bertrand O. (1999). Oscillatory gamma activity in humans and its role in object representation. *Trends Cogn. Sci.* 3 151–162. 10.1016/S1364-6613(99)01299-1 10322469

[B114] Tiitinen H. T. Sinkkonen J. Reinikainen K. Alho K. Lavikainen J. Näätänen R. (1993). Selective attention enhances the auditory 40-Hz transient response in humans. *Nature* 364 59–60. 10.1038/364059a0 8316297

[B115] Torres M. E. Colominas M. A. Schlotthauer G. Flandrin P. (2011). “A complete ensemble empirical mode decomposition with adaptive noise,” in *2011 IEEE International conference on acoustics, speech and signal processing (ICASSP)*, (Piscataway, NJ: IEEE), 4144–4147. 10.1109/ICASSP.2011.5947265

[B116] Tsai C. C. Liang W. K. (2021). Event-related components are structurally represented by intrinsic event-related potentials. *Sci. Rep.* 11:5670. 10.1038/s41598-021-85235-0 33707511 PMC7970958

[B117] Turchi J. Chang C. Ye F. Q. Russ B. E. Yu D. K. Cortes C. R. (2018). The basal forebrain regulates global resting-state fMRI fluctuations. *Neuron* 97 940–952.e4. 10.1016/j.neuron.2018.01.032 29398365 PMC5823771

[B118] van der Zande J. J. Gouw A. A. van Steenoven I. Scheltens P. Stam C. J. Lemstra A. W. (2018). EEG characteristics of dementia with Lewy bodies, Alzheimer’s disease and mixed pathology. *Front. Aging Neurosci.* 10:190. 10.3389/fnagi.2018.00190 30018548 PMC6037893

[B119] van der Zande J. J. Gouw A. A. Van Steenoven I. Van De Beek M. Scheltens P. Stam C. J. (2020). Diagnostic and prognostic value of EEG in prodromal dementia with Lewy bodies. *Neurology* 95 E662–E670. 10.1212/WNL.0000000000009977 32636325

[B120] van Nifterick A. M. Mulder D. Duineveld D. J. Diachenko M. Scheltens P. Stam C. J. (2023). Resting-state oscillations reveal disturbed excitation–inhibition ratio in Alzheimer’s disease patients. *Sci. Rep.* 13:7419. 10.1038/s41598-023-33973-8 PMC10164744 37150756

[B121] van Straaten E. C. W. Scheltens P. Gouw A. A. Stam C. J. (2014). Eyes-closed task-free electroencephalography in clinical trials for Alzheimer’s disease: An emerging method based upon brain dynamics. *Alzheimers Res. Ther.* 6:86. 10.1186/s13195-014-0086-x 25621017 PMC4304266

[B122] Wang J. Zhang H. Y. Tang X. C. (2009). Cholinergic deficiency involved in vascular dementia: Possible mechanism and strategy of treatment. *Acta Pharmacol. Sin.* 30 879–888. 10.1038/aps.2009.82 19574993 PMC4006646

[B123] Wang Z. Liu A. Yu J. Wang P. Bi Y. Xue S. (2023). The effect of aperiodic components in distinguishing Alzheimer’s disease from frontotemporal dementia. *Res. Sq.* [Preprint]. 10.21203/rs.3.rs-2915225/v1 PMC10828513 38110590

[B124] Whitham E. M. Lewis T. Pope K. J. Fitzgibbon S. P. Clark C. R. Loveless S. (2008). Thinking activates EMG in scalp electrical recordings. *Clin. Neurophysiol.* 119 1166–1175. 10.1016/j.clinph.2008.01.024 18329954

[B125] Whittington M. A. Cunningham M. O. LeBeau F. E. N. Racca C. Traub R. D. (2011). Multiple origins of the cortical gamma rhythm. *Dev. Neurobiol.* 71 92–106. 10.1002/dneu.20814 21154913

[B126] Widmann A. Schröger E. (2012). Filter effects and filter artifacts in the analysis of electrophysiological data. *Front. Psychol.* 3:233. 10.3389/fpsyg.2012.00233 22787453 PMC3391960

[B127] Wu L. Chen Y. Zhou J. (2014). A promising method to distinguish vascular dementia from Alzheimer’s disease with standardized low-resolution brain electromagnetic tomography and quantitative EEG. *Clin. EEG Neurosci.* 45 152–157. 10.1177/1550059413496779 24214287

[B128] Wu Z. Huang N. E. (2009). Ensemble empirical mode decomposition: A noise-assisted data analysis method. *Adv. Adapt. Data Anal.* 1 1–41. 10.1142/S179353690900004

