# Supplementary material for: A holo-spectral EEG analysis provides an early detection of cognitive decline and predicts the progression to Alzheimer’s disease
Source: Front Aging Neurosci. 2023 Aug 22;15:1195424. doi: 10.3389/fnagi.2023.1195424 (PMC10477374; doi:10.3389/fnagi.2023.1195424)
Supplement: Supplementary file 1 [file Data_Sheet_1.PDF]

Supplementary materials

A Holo-Spectral EEG Analysis Provides Early Detection of Cognitive Decline and Predicts Progression to Alzheimer's Disease

Kwo-Ta Chu, Weng-Chi Lei, Ming-Hsiu Wu, Jong-Ling Fuh, Shuu-Jiun Wang, Isobel T. French, Wen-Sheng Chang, Chi-Fu Chang, Norden E. Huang, Wei-Kuang Liang, and Chi-Hung Juan*

*** Correspondence:** Corresponding Author: chijuan@cc.ncu.edu.tw

Supplementary figures and tables

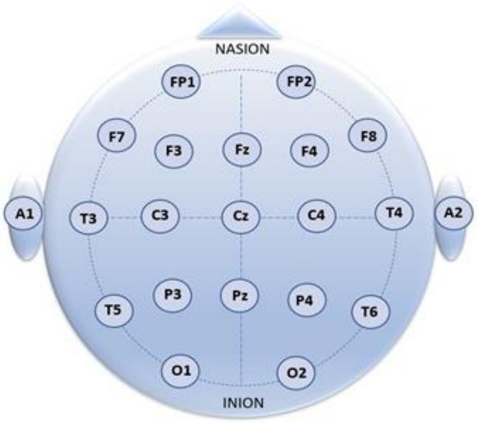

Brain region	Channel name
Frontal	FP1, FP2, F7,F3,Fz, F4, F8
Central	C3, Cz,C4
Parietal	P3,Pz,P4
Temporal	T3,T5,T6,T4
Occipital	O1,O2

S1 Montage and channel names of EEG electrodes with their topographical channel names categorized into five brain regions. The placement of 19 electrodes uses an electrode cap with 2 reference electrodes (A1& A2) attached to the mastoid according to the standardized 10-20 system. The 19 electrodes are labeled as Fp1, Fp2, F7, F3, Fz, F4, F8, T3, C3, Cz, C4, T4, T5, P3, Pz, P4 ,T6 ,O1 and O2. Based on the International 10-20 system of electrode placement, EEG 19 channels can be categorized into five brain regions. Frontal: Fp1, Fp2, F7, F3, Fz, F4, F8; Central: C3, Cz, C4; Parietal: P3, Pz, P4; Temporal: T3, T5, T6, T4; Occipital: O1, O2.

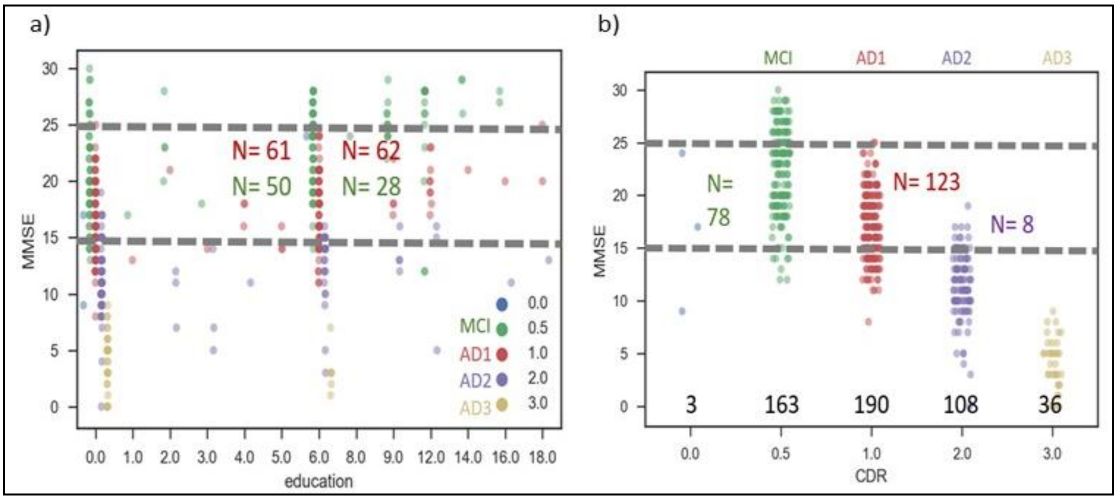

S2 Division of subject groups based on MMSE and CDR scores: a) *Educational level plays a significant role in MCI. The group scatter plot is based on educational level and MMSE score, where the clustering effect is due to the schooling system; zero for no education, six for elementary school, nine for junior high school, and twelve for senior high school. Taking six years of education as the cutoff, we see that 61 AD1 patients receive less than six years, and 62 receive more than six years. Contrarily, 50 MCI patients receive less than six years of education, and 28 receive more than six years. With increasing education levels, the number of patients with dementia is relatively reduced. At the same time, apparent clustering phenomena are seen in patients receiving little or no education, followed by those receiving six years of education with significant clustering, with less obvious clustering in those with 9 and 12 years of education. Therefore, patients with significant cognitive impairment have a similar chance of suffering from mild AD between the above six-year and below six-year subgroups, according to the MMSE system. Still, the possibility of MCI is higher in the below-six-year subgroup, b) The group scatter plot is based on the relationship between MMSE and CDR; the MMSE scoring system defines a value less than 24 as MCI and less than 18 as severe cognitive impairment* (Folstein et al., 1975). *According to the CDR, MMSE scores between 15 and 25 include 78 cases of MCI, 123 cases of mild AD, and 8 cases of moderate AD. Therefore, lone CDR categorization is insufficient; incorporating the MMSE system better discriminates AD from MCI. We chose an MMSE score of 25 and a CDR score to separate MCI and mild AD in the first part of our EEG group-level analysis. Still, we only used a CDR of 0.5 with a centralized MMSE score near 25 to include more participants in the MCI longitudinal cohort.*

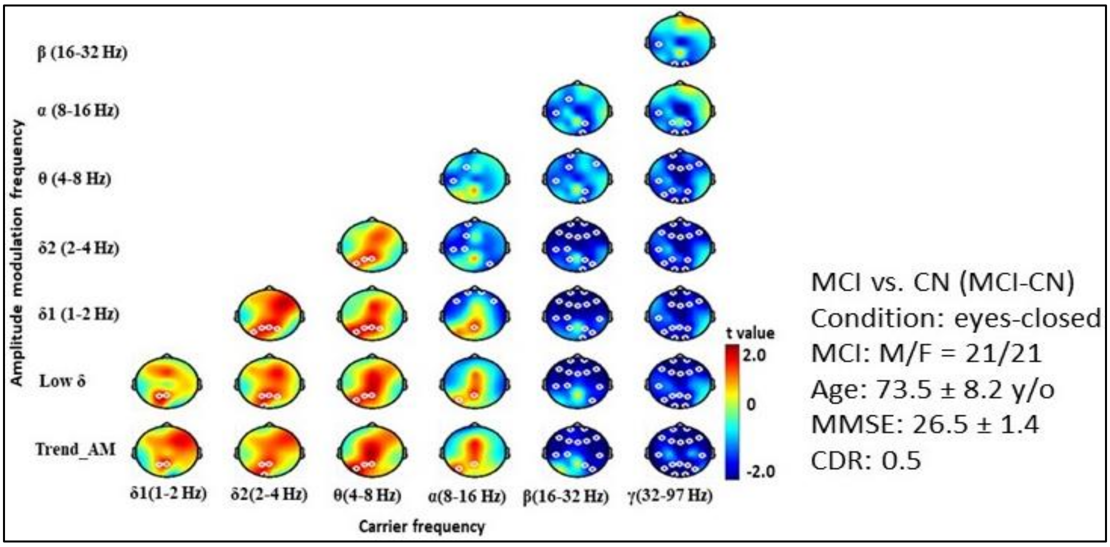

S3 Contrasted HHSA EEG between MCI and CN (MCI-CN, EC condition): Increasing amplitude modulation energy (AM) of lower-frequency oscillations (LFO; delta and theta bands) with decreasing AM energy of higher-frequency oscillations (HFO; beta and gamma bands) are observed. As a transitional zone, the alpha band revealed increasing AM energy over posterior brain regions with reduced AM energy over anterior brain regions. The x-axis denotes the carrier frequency, and the y-axis depicts the amplitude modulation frequency. The frequency bin is on a dyadic scale (2^{-1}, 2^{0}, 2^{1}, 2^{2}, 2^{3}, etc.), except for the gamma band, where the upper limit of the gamma band is 100 Hz based on the Nyquist rule. The color bar denotes t-statistics ranging from blue (-2) to red (+2). The white circles indicate the contrast on those EEG channels is a statistically significant difference ($p<0.05$, cluster permutation test, two-tailed, cluster distance: 70mm, permutation: 5000).

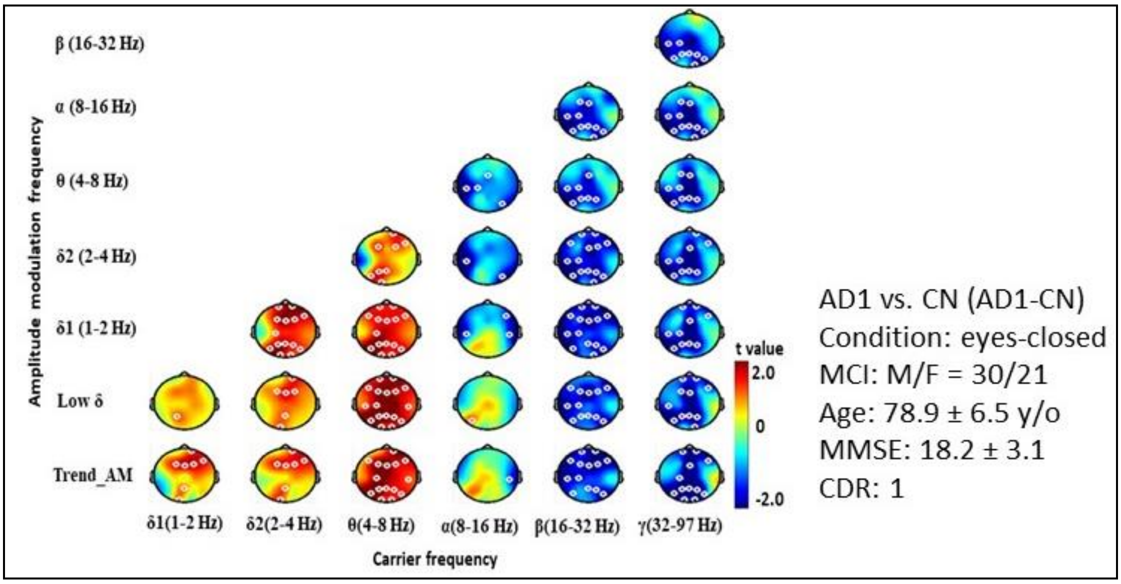

S4 Contrasted HHSA rsEEG between AD1 and CN: Increasing AM energy of LFO (delta and theta bands) coupled with decreasing AM energy of HFO (beta and gamma bands) are seen globally. In alpha oscillations, reducing AM power was sparsely scattered in several brain regions, whereas increasing AM power was in the left temporal area. The annotation is the same as in S3.

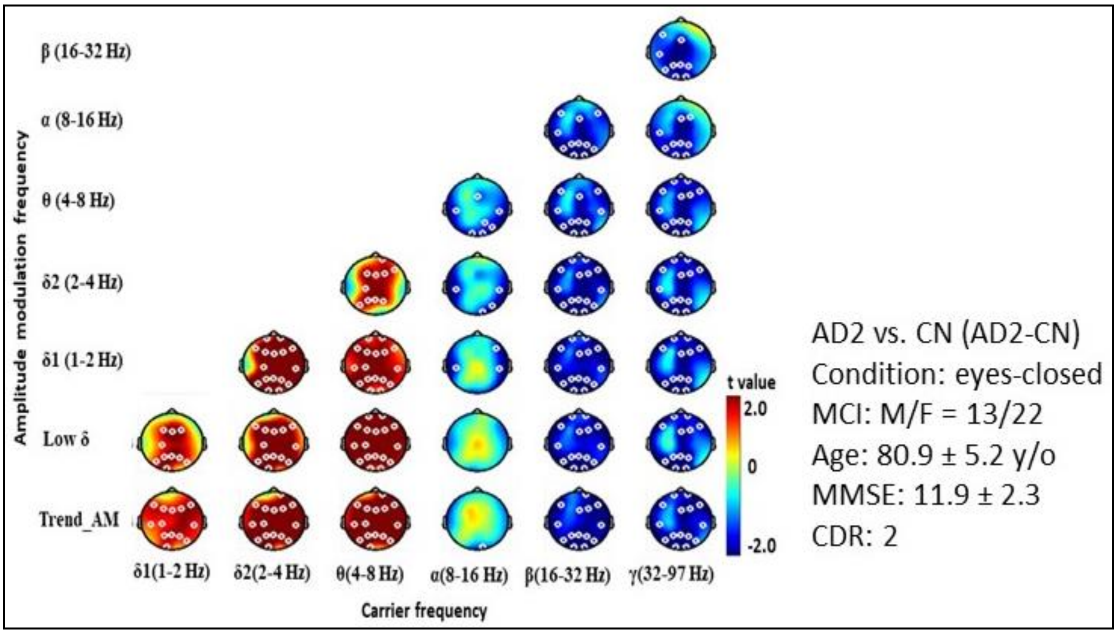

S5 Contrasted HHSA EEG between AD2 and CN: Increasing AM power of LFO (delta and theta bands) with broadly decreasing AM power of HFO (alpha, beta, and gamma bands) are observed. The alpha band's negative modulating trend scattered sparsely across the frontal, temporal, parietal, and occipital regions. The annotation is the same as in S3.

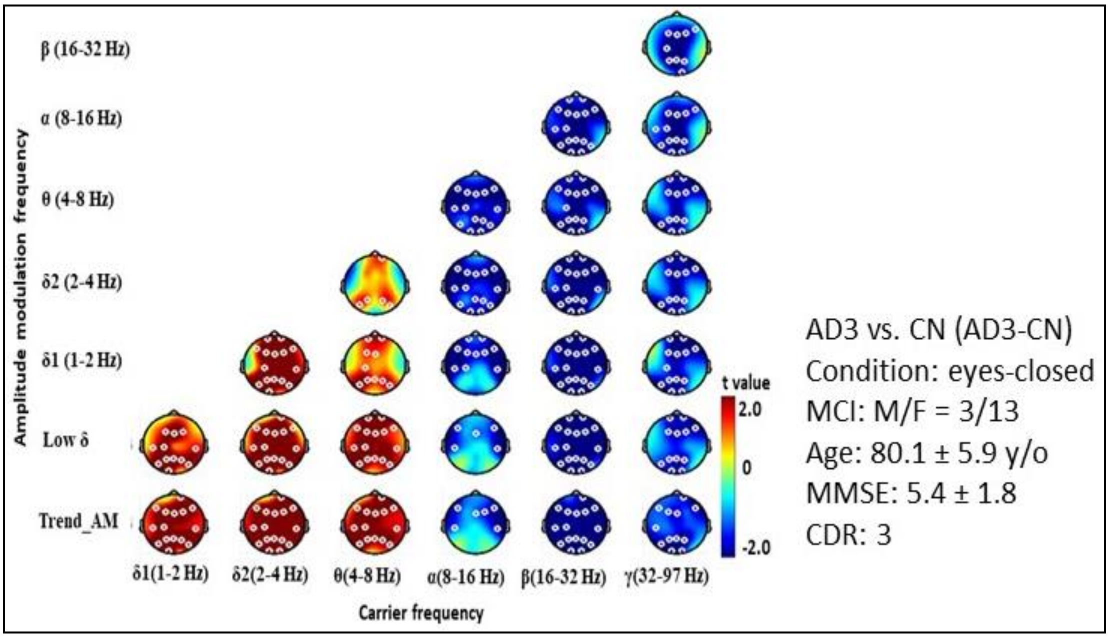

S6 Contrasted HHSA EEG between AD3 and CN: Increasing AM energy of LFO (delta and theta bands) with broadly decreasing AM energy of HFO (alpha, beta, and gamma bands) are seen. Though the LFO AM energy density positive modulating trends were broadly distributed, theta AM power modulating theta carrier frequency was sparsely dispersed in anterior brain regions. The annotation is the same as in S3.

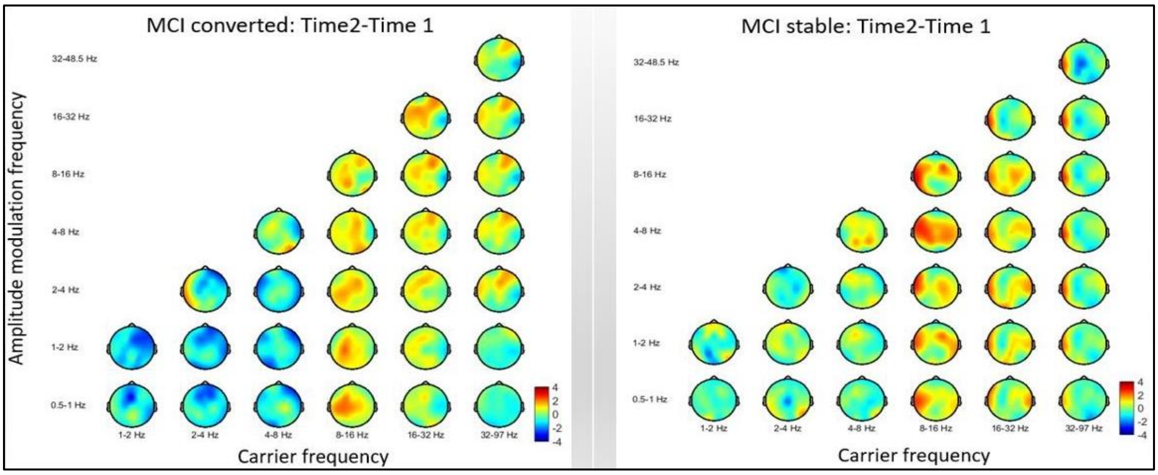

S7 Intra-group comparisons within 3-year longitudinal rsEEGs in MCI-converted and MCI-stable subgroups. Left: The MCI-C within-group analysis shows a trend of increasing AM power in the alpha and beta bands, with decreasing AM power in theta and delta bands. Right: The MCI-S within-group analysis showed a trend of increasing AM energy in the alpha band. However, no statistically significant contrasts are seen between electrodes ($p<0.05$, CBnPP with 5000 permutations, two-tailed; max distance (cluster) = 70mm)

HHSA-extracted features	HHT-extracted features	Windowed FFT-extracted features
Fp1, fam 0.5-1 Hz, fc 2~4 Hz (δ2)	Fp2, fc 0.5~1 Hz (L*f*)	F3, fc 12.5~30 Hz (β)
P4, fam 2~4 Hz, fc 2~4 Hz (δ2)	F3, fc 4~8 Hz (θ)	F4, fc 0.5~4 Hz (δ)
O1, fam 0.5~1 Hz, fc 2~4 Hz (δ2)	C4, fc 1~2 Hz (δ1)	C3, fc 30.5~80 Hz (γ)
O1, fam 0.5~1 Hz, fc 4~8 Hz (θ)	O1, fc 4~8 Hz (θ)	O1, fc 4.5~8 Hz (θ)
O2, fam 16~32 Hz, fc 16~32 Hz (β)	O2, fc 0.5~1 Hz (L*f*)	O1, fc 8.5~12 Hz (α)
F8, fam 2~4 Hz, fc 8~16 Hz (α)	T3, fc 4~8 Hz (θ)	T3, fc 0.5~4 Hz (δ)
T3, fam 2~4 Hz, fc 16~32 Hz (β)	Fz, fc 8~16 Hz (α)	T5, fc 12.5~30 Hz (β)

S8 Comparisons among features extracted from three analytic methods (HHSA, HHT, FFT). The feature codes in HHSA-extracted include channel name, AM frequency (fam), and carrier frequency (fc), whereas HHT-extracted and Windowed FFT-extracted only yield channel name and carrier frequency.

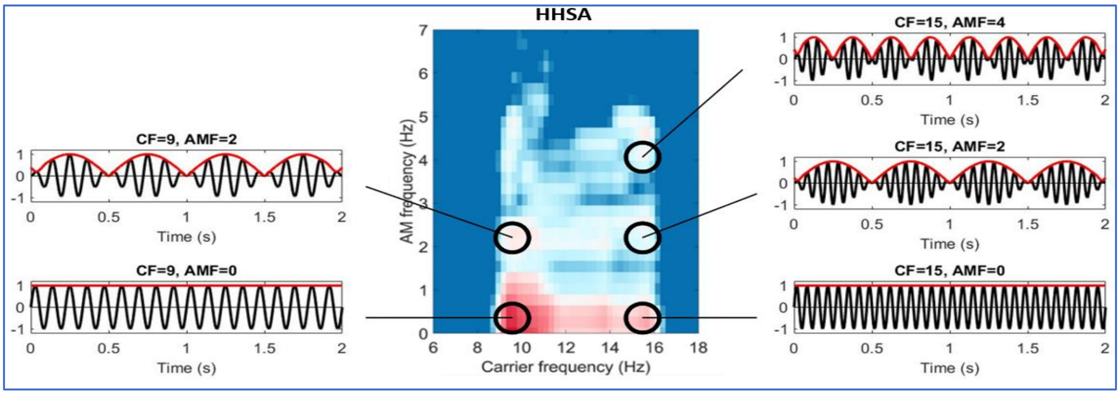

S9 This diagram shows the simulated waveforms with different numeric combinations of carrier frequency (i.e., black waves) and amplitude modulation frequency (i.e., red waves), which are displayed using the Holo-Hilbert spectral analysis (HHSA). CF denotes carrier frequency as the x-axis, and AMF represents amplitude modulation frequency as the y-axis. The red color density reveals that the AM power of CF is collapsed across the time domain

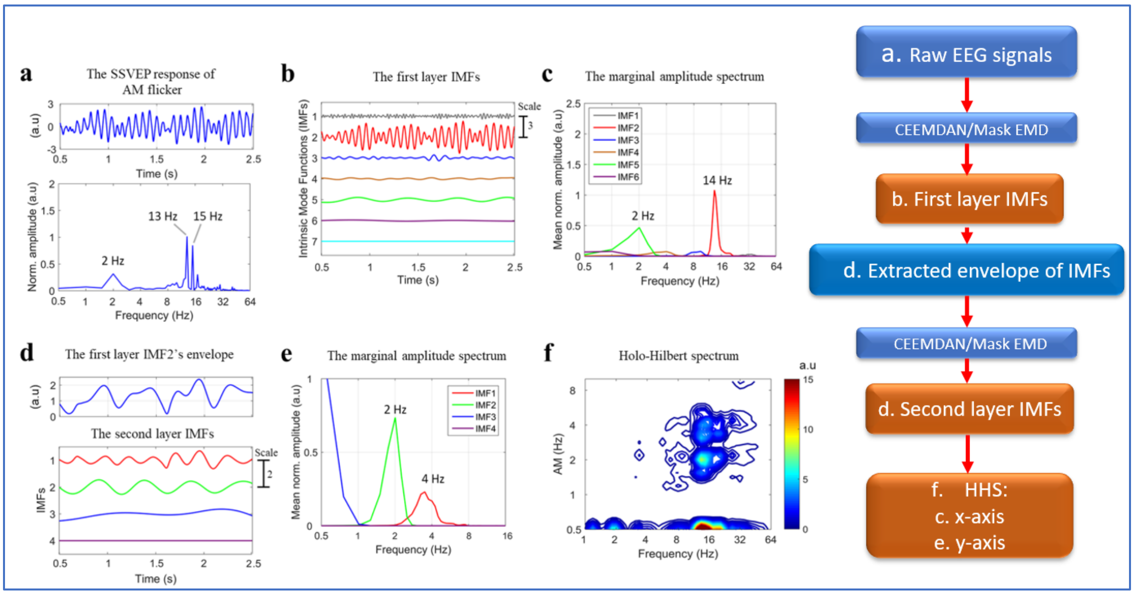

S10 The Holo-Hilbert Spectrum of SSVEP response induced by AM flicker (2|14 Hz) at Oz channel. (a) The SSVEP response in the time domain and FFT-based frequency domain. The power spectrum shows peaks at frequencies of 13 Hz, 15 Hz, 2 Hz, and harmonic of 28 Hz. (b) The first masking EMD yields the first layer of 7 IMFs. IMF2 and IMF5 correspond to the carrier frequency of 14 Hz and AM frequency of 2 Hz, respectively. (c) The marginal amplitude spectrum of the HHT reveals high-resolution peaks at 2 Hz and 14 Hz, compared with the FFT power spectrum. (d) Applying the second masking EMD to the envelope of the first layer IMF2 yields the second layer of 4 IMFs. (e) The marginal amplitude spectrum of the second layer IMFs of HHT shows peaks at 2 Hz and 4 Hz, which are the AM frequencies. (f) The HHSA of SSVEP response induced by AM flicker shows a two-dimensional frequency spectrum, which displays power increment at 2 Hz and 16 Hz (stimulus frequency) with amplitude modulation ranging from 1 to 4 Hz. The x-axis denotes carrier frequency, and the y-axis represents AM frequency. At AM frequency of 0.5 Hz, the x-axis is the summed power of carrier frequency across time (reproduced from Nguyen et al., 2019 *with permission).*

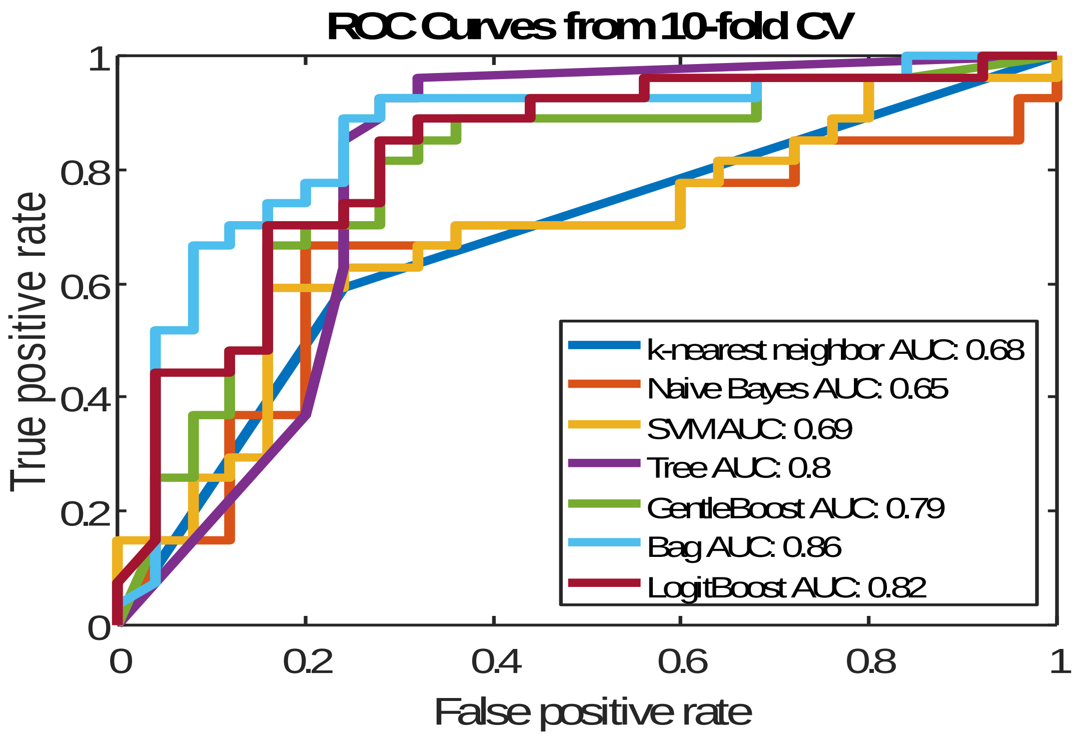

S11 Seven algorithms underwent 10-fold cross-validation, which yielded receiver operating characteristic (ROC) curves with area under the ROC curve (AUC) values. Applying extracted features to fit the machine learning algorithm with 10-fold cross-validation in the training stage yielded the ROC curve and AUC values. In the testing stage, we tested the untrained dataset and output the performance matrices in main text Tables 4,5,6, and 7.

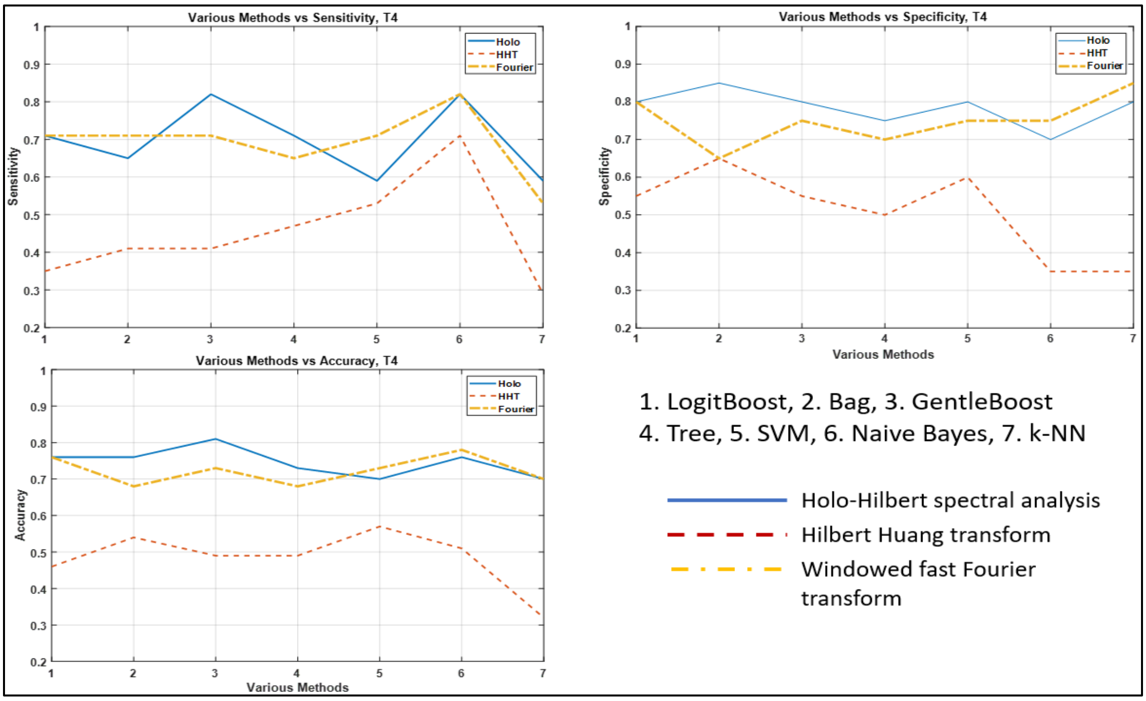

S12 Visualization of performance metrics of classification algorithms that deployed features extracted from the rsEEG comparison between MCI and CN using various analytic methods (derived from main text Table 4). The HHSA-based feature extraction outperformed other methods, in which the GentleBoost algorithm surpassed other classifiers with a sensitivity of 82%, specificity of 80%, and an accuracy of 81%, exhibiting the best performance metrics among the three analytic methods

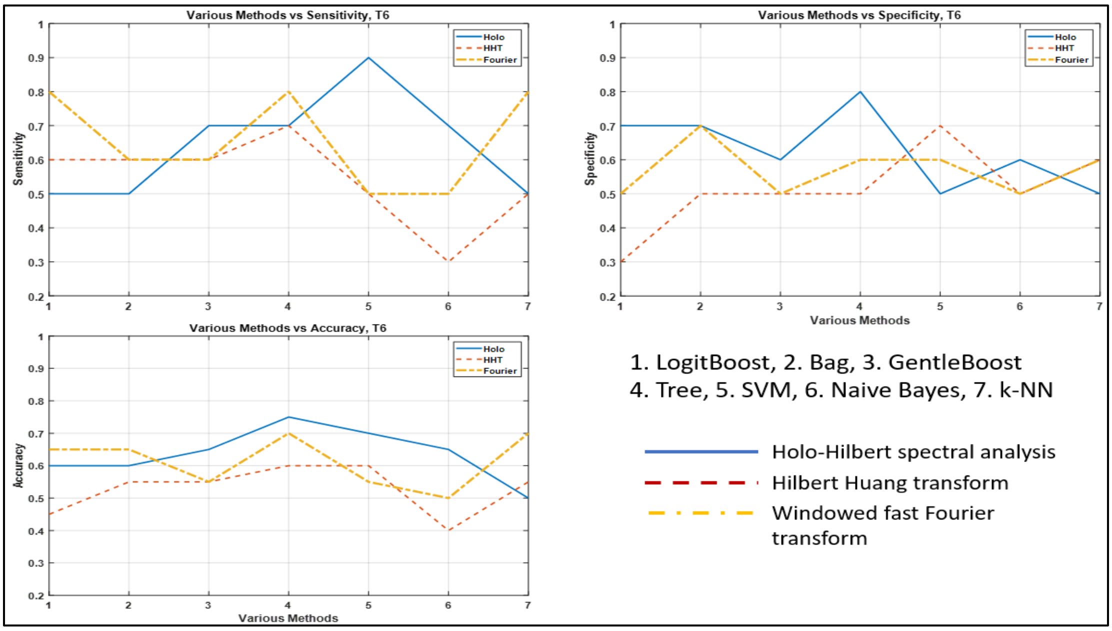

S13 Visualization of performance metrics of prediction algorithms deploying features extracted from baseline rsEEG comparison between MCI-C and MCI-S using various analytic methods). The HHSA-based feature extraction surpassed other methods, where the Decision Tree in the prediction algorithms of MCI conversion gave the best accuracy of 75% and specificity of 80%, whereas SVM output the best sensitivity of 90%.

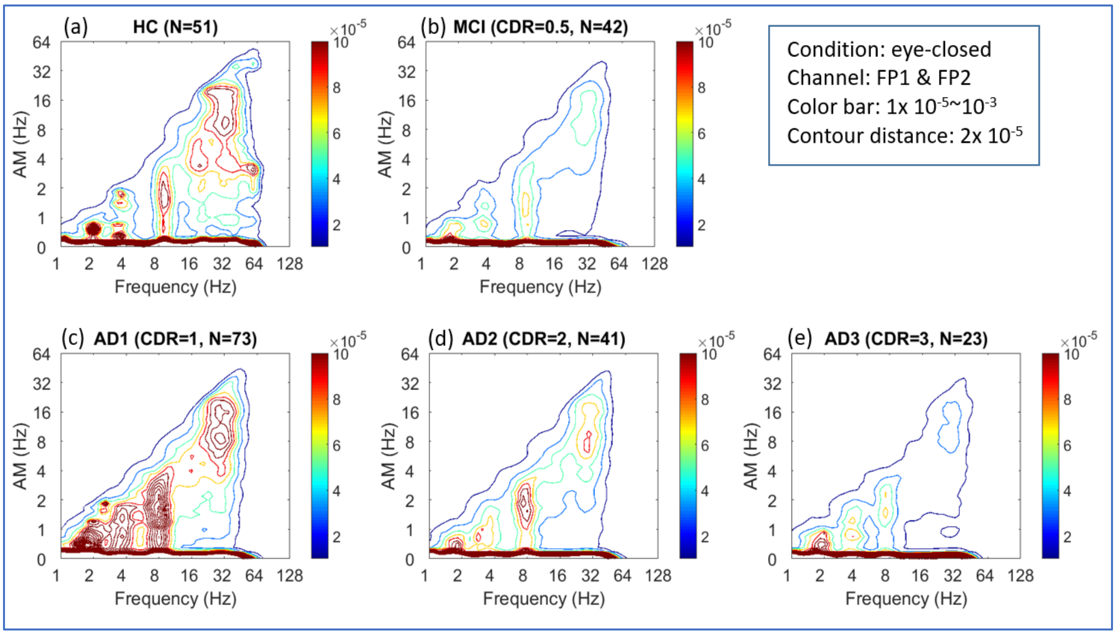

S14 Individual group-level HHSA rsEEG on averaged FP1 and FP2 channels revealed an increment in energy density of lower-frequency brain oscillations (i.e., delta and theta bands) in all AD subgroups, whereas decrement in energy density of higher-frequency brain oscillations (i.e., beta and gamma) in MCI and other AD subgroups, but not in AD1. Panel (a) shows alpha peak frequency at 10 Hz in the healthy control (HC) group, with 9 Hz in MCI (panel b) and 8 Hz in other AD subgroups (panels c to e), accompanied by the above-mentioned altered oscillations pattern, indicating an EEG rhythm slowing phenomenon. In this AM-FM energy map, at the AM frequency below 0.5 Hz, the FM power is the summed amplitude over time, which is the trend of AM frequency, an aperiodic power activity ((Nguyen et al., 2019: Huang et al., 2016).

\

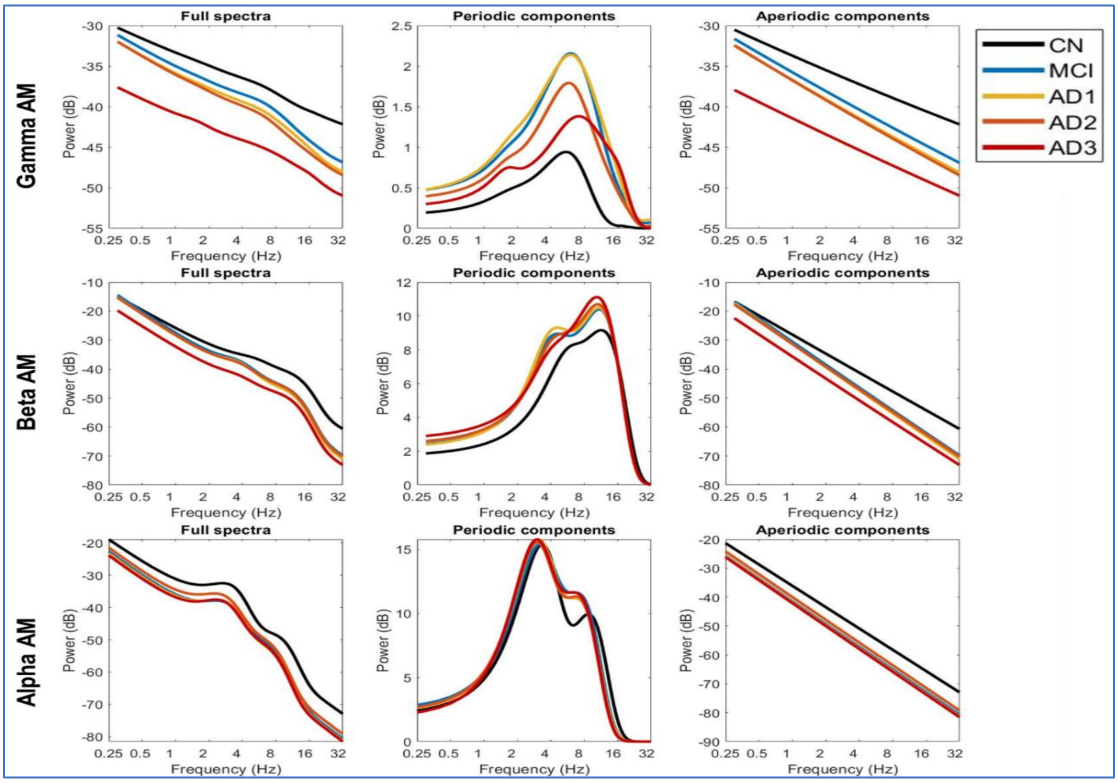

S15 Illustration of oscillatory activity in amplitude modulation by HHSA-based AM spectra of corresponding gamma fc, beta fc, and alpha fc in all groups, fitted FOOOF algorithm (Donoghue et al., 2020). *The amplitude modulation corresponds to gamma fc, beta fc, and alpha fc, designated as gamma AM, beta AM, and alpha AM, respectively. Similarly, we have performed FOOOF analysis to gamma (IMFs 1 and 2), beta (IMF 3), and alpha (IMF 4) AM, the results are illustrated in S15. The AM full spectra of all groups (Fig. 15 left column) decomposed by multitaper are fitted in the FOOOF algorithm, which yields periodic (Fig. 15 middle column) and aperiodic components (Fig. 13 right column). The periodic spectra of gamma AM still reveal oscillatory activities ranging from 2 to 32 Hz with a central frequency of 7-8 Hz. The periodic spectra of beta AM display oscillatory characteristics between 2 and 32 Hz with two peaks across the AD continuum. The aperiodic exponents exhibit an increase in all groups. The periodic spectra of alpha AM show oscillatory patterns between 1 and 16 Hz with two peaks at 3 and 8 Hz in the AD continuum, but no difference in aperiodic exponent between groups.*

*Statistically speaking, for gamma AM, the power of periodic component in the control group is different from the AD groups (F(4,200)=7.09, p<2.34*10-5). The offset of aperiodic components decreases with AD progression (F(4,200)=13.63, p<1*10-5), and the exponents are larger for the MCI, AD1, and AD2 groups when compared with CN and AD3 groups (F(4,200)=8.02, p<1*10-5). For beta AM, both the power of periodic components (F(4,200)=61.5, p<1*10-5) and the aperiodic exponents (F(4,200)=89.57, p<1*10-5) increase with AD progression, whereas the offset decreases with AD progression (F(4,200)=6.88, p=3.30*10-5). For alpha AM, the power of periodic components decreases (F(4,200)=4.73, p=0.0011). The exponent (F(4,200)=10.7, p<1*10-5) and the offset (F(4,200)=24.57, p<1*10-5) also show differences between the control and AD groups. In sum, our data demonstrate that the AM of different IMFs exhibits a 1/f-like power spectrum, even when analyzed with conventional methods such as multitapers. Therefore, there is a true oscillatory activity in the AM of different IMFs.*

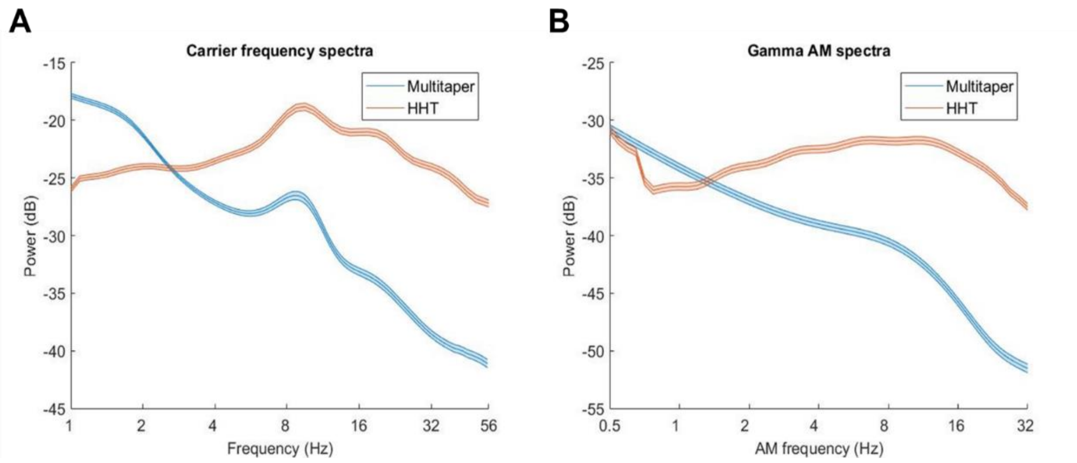

S16. Comparison of spectra using multitapers and HHT: Shaded areas denote the 95% CI. (A) The averaged multitaper spectrum for the dataset of this study shows a typical 1/f structure, with a peak in the alpha band (blue line). In contrast, the HHT spectrum does not have the 1/f shape (red line). As a result, there is more oscillatory power in the gamma band (<32 Hz). (B) The power spectrum of gamma AM (from the envelopes of IMFs 1 and 2) shows that there is a substantial amount of low-frequency modulatory power for gamma activity, as revealed by HHT (red line). The mismatch between multitaper and HHT spectra arises from the nonlinear amplitude modulation of gamma power.

Machine learning	Parameters	Ensemble learning	Parameters
Logistic Regression (LR)	Solver=‘ibfgs’,max_iter =2000	Bagging (Bagged)	base_estimator= Decision Tree Classifier; n_estimators=100; random_state=7
Linear Discriminant Analysis (LDA)	Default settings: solver= ‘svd’	Ada Boost (Ada)	base_estimator =None(Decisioni Tree Classifier); learning_rate=1; n_estimators=100; random_state=7
K-Neighbors Classifier (KNN)	Default settings: n_neighbors=5	Random Forest (RF)	n_estimators=100
Classification & Regression Tree (CART)	Default settings:criterion =‘gini’; splitter =‘best’;max_depth=‘non’;min_samples_split=2	Extra Trees (ETC)	n_estimators=100
Naïve Bayes Classifiers (NB)	Default settings:	Gradient Boosting (SGB)	N_estimators=100; random_state=7’; loss=‘deviance’; learning_rate=0.1; subsample=1
Support Vector Machine (SVM)	C=1; Kernel=‘rbf’; gamma=‘auto’	Voting Classifier	Logistic Regression; Decision Tree Classifier; SVC (gamma=‘auto’)

S17 Parameters of algorithms in Machine learning/Ensemble learning

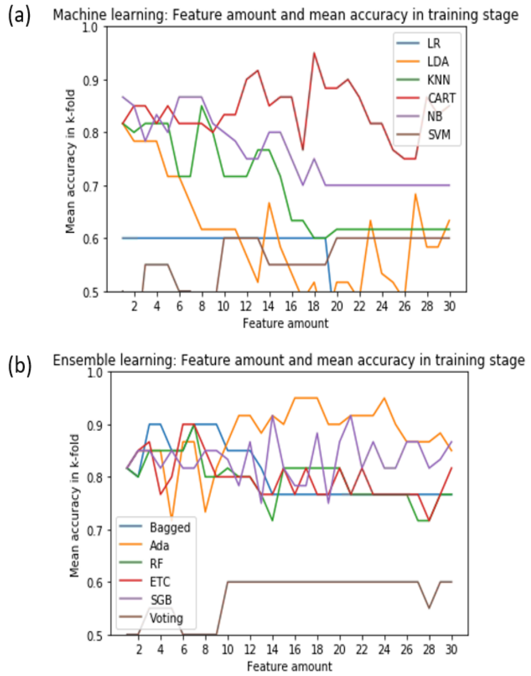

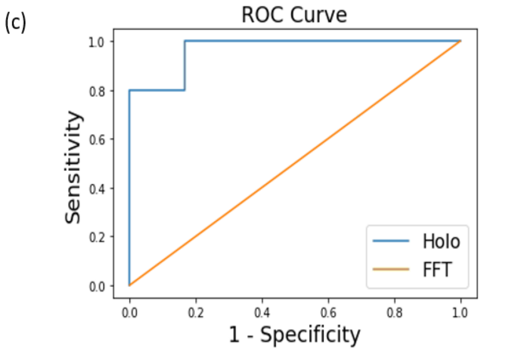

(d)

Group	Total	Train	Test
MCI-stable	18	12	6
MCI -concert	15	10	5

Test result	ACC	AUC	Sensitivity	Specificity
HHSA	0.82	0.97	0.83	1
FFT	0.55	0.50	1	0

S18 Illustration of deploying various feature amounts influences the classifier's performance in the training stage and testing stage for prediction of MCI conversion. For abbreviation, see S17. Holo-Hilbert Spectral Analysis, FFT: fast Fourier transform

S19 A confusion matrix is a table used to evaluate the performance of a classification model. It shows the number of true positives, false positives, true negatives, and false negatives for each class in the dataset.

- *The formulas for precision and recall are:*

precision *= TP / (TP + FP)*

recall *= TP / (TP + FN)*

where TP is the number of true positives, FP is the number of false positives, and FN is the number of false negatives, TN is the number of true negatives.

where ***precision*** *is the number of true positives divided by the sum of true positives and false positives, and* ***recall*** *is the number of true positives divided by the sum of true positives and false negatives.*

- *The formula for calculating the accuracy of a classification model using a confusion matrix is:*

accuracy *= (TP + TN) / (TP + TN + FP + FN).*

- *The* ***F1 score*** *is a measure of a model's accuracy that combines precision and recall. It is the harmonic mean of precision and recall and is defined as:*

F1 score *= 2 * (precision * recall) / (precision + recall)*

An example of how to calculate precision and recall using a confusion matrix:

Suppose we have a binary classification problem with two classes, A and B. We have a dataset with 100 examples, 80 of which are class A and 20 of which are class B. We train a classification model on this dataset and obtain the following confusion matrix:

Predicted A	*Predicted A*	*Predicted B*
Actual A	*70*	*10*
Actual B	*20*	*0*

The precision of the model for class A is:

precision = TP / (TP + FP) = 70 / (70 + 20) = 0.78

Domain of signals	Decomposition method	FOOOF algorithm	Figure illustration
Carrier frequency	multitaper	Periodic and aperiodic components	Main text Figure 3
Amplitude modulation	HHSA, then multitaper	Periodic and aperiodic components	Supplementary materials: S15

S20 Summary of carrier frequency and AMs subjected to each decomposition process and figure illustration.

The sequence of our data processing, including carrier frequency and AM subjected to each decomposition method and FOOOF algorithm, which display the periodic and aperiodic components in text figure and supplementary materials, is shown in S20.

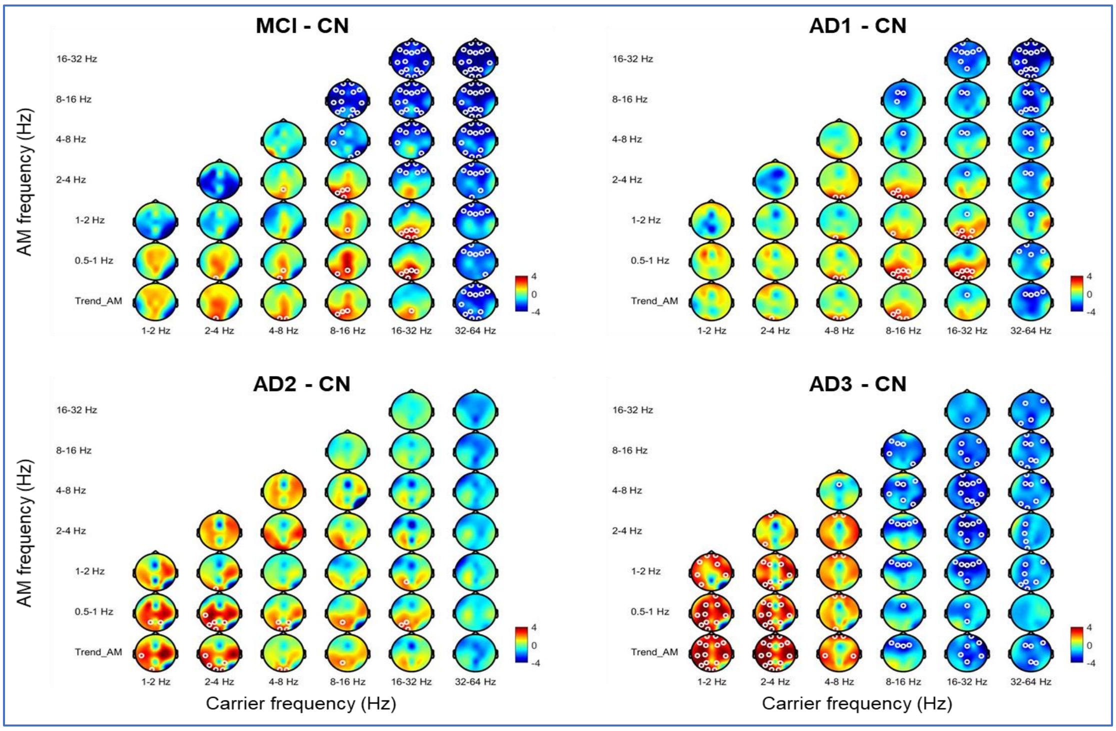

S21 Comparisons of eye-closed EEG between AD groups and the control group, with age as the covariate: Progressive increment in AM power of LFO (delta and theta bands) coupled with decrement in AM power of HFO existed across MCI to AD continuum. Age is an essential factor contributing to disease onset of AD, and was a confounding factor in our study. Thus, we used age as the covariate to reanalyze our group-level comparison across MCI to the AD continuum with CN. In general, the effect size amid group difference decreased after correction. However, stage-wise oscillatory pattern changes were still observed as seen in the unadjusted comparison. Progressive increment in AM power of LFO coupled with decrement in AM power of HFO existed across MCI to AD subgroups, similar to what was observed in Figure 2 of the main text, with some increases in posterior beta AM power in MCI-CN and AD1-CN contrast HHSA. The MCI-CN contrast HHSA reveals sparse increment in AM power of LFO (i.e., delta and theta) in posterior brain regions with decrement in AM power of HFO (i.e., beta and gamma) globally. The increment in posterior AM power broadens from alpha to beta bands, which is consistent with the MCI-stable group in the longitudinal MCI cohort, suggesting a compensatory augmented alpha and beta AM power in MCI patients.

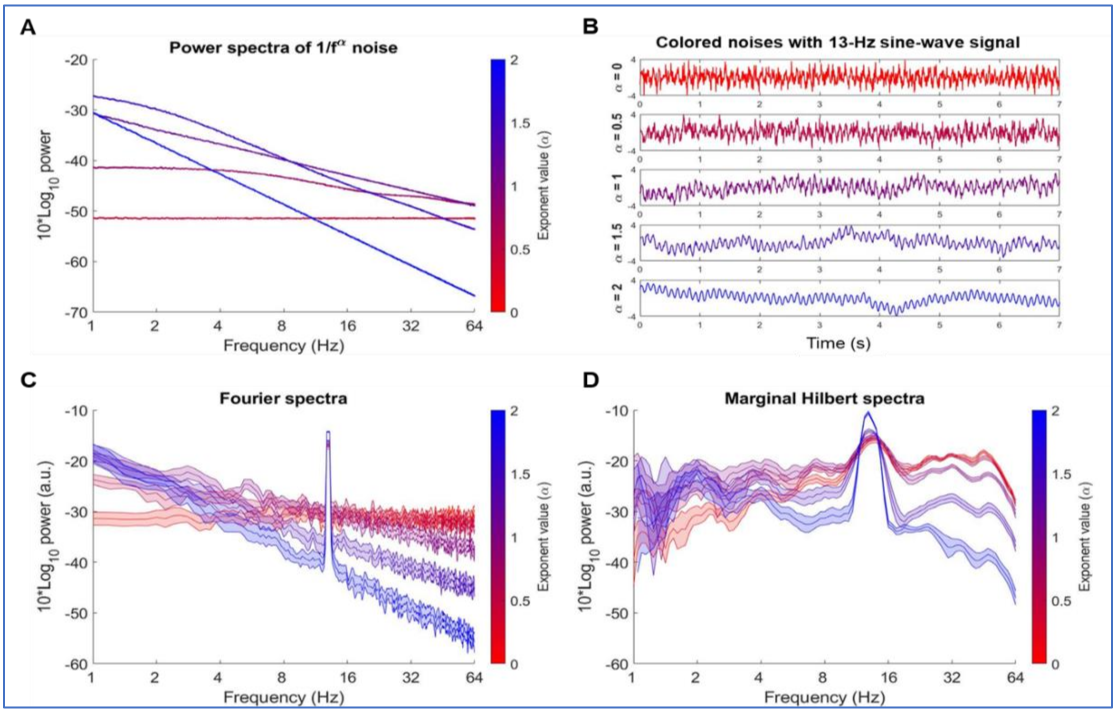

S22 Comparison of 13-Hz sine wave under different colored noises: (A) The Fourier power spectra of different colored noises; 0 (white noise), 0.5, 1 (pink noise), 1.5 (neuronal avalanche), and 2 (Brownian noise). (B) Exemplar signals with different colored noises. (C) The Fourier power spectra of the simulated signals. Shaded areas denote the 95% confidence intervals of 19 repetitions. (D) The HHT power spectra of the same signals. Lower frequency power shows no difference between various exponents, indicating no inflation by exponents when using the HHT method. However, the higher-frequency power reveals decrement with exponent increment.

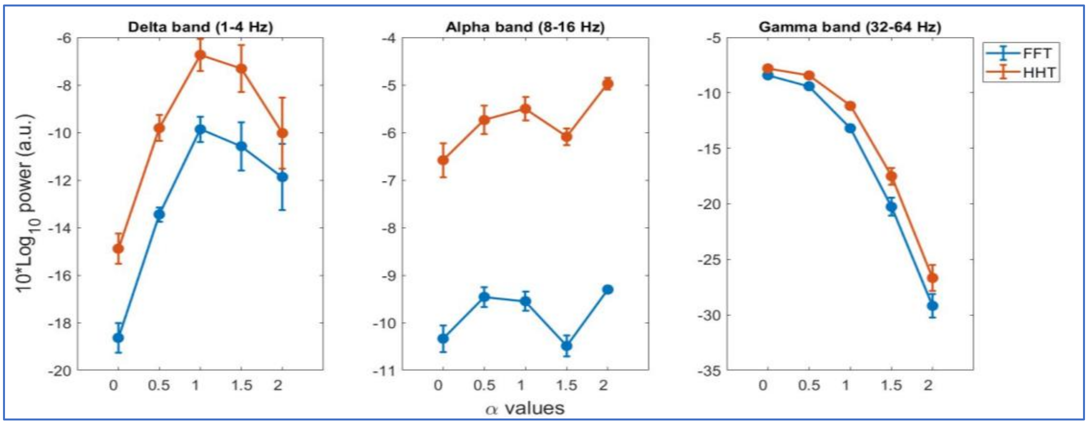

S23 Comparisons of FFT and HHT power spectra under different colored noises: Error bars denote the 95% confidence intervals. The power increases with steeper exponents. Comparisons of FFT and HHT power spectra of the simulated 13 Hz sine wave using graded exponent noise show increment in power with exponent increases in lower-frequency bands. HHT decomposition shows higher power spectra in delta and alpha bands, suggesting a higher signal-to-noise ratio. However, gamma power decreases with exponent increment, with no difference between the two methods.

References:

Donoghue, T., Haller, M., Peterson, E. J., Varma, P., Sebastian, P., Gao, R., Noto, T., Lara, A. H., Wallis, J. D., Knight, R. T., Shestyuk, A., & Voytek, B. (2020). Parameterizing neural power spectra into periodic and aperiodic components. *Nature Neuroscience*, *23*(12), 1655–1665. https://doi.org/10.1038/s41593-020-00744-x

Folstein, M. F., Folstein, S. E., & McHugh, P. R. (1975). "Mini-mental state" A practical method for grading the cognitive state of patients for the clinician. *Journal of Psychiatric Research*, *12*(3), 189–198. https://doi.org/10.1016/0022-3956(75)90026-6

Huang, N. E., Hu, K., Yang, A. C. C., Chang, H. C., Jia, D., Liang, W. K., Yeh, J. R., Kao, C. L., Juan, C. H., Peng, C. K., Meijer, J. H., Wang, Y. H., Long, S. R., & Wu, Z. (2016). On holo-Hilbert spectral analysis: A full informational spectral representation for nonlinear and non-stationary data. *Philosophical Transactions of the Royal Society A: Mathematical, Physical and Engineering Sciences*, *374*(2065). https://doi.org/10.1098/rsta.2015.0206

Huang, N. E., Shen, Z., Long, S. R., Wu, M. C., Shih, H. H., Zheng, Q., Yen, N.-C., Tung, C. C., & Liu, H. H. (1998). The empirical mode decomposition and the Hilbert spectrum for nonlinear and non-stationary time series analysis. *Proceedings of the Royal Society of London. Series A: Mathematical, Physical and Engineering Sciences*, *454*(1971), 903–995. https://doi.org/10.1098/rspa.1998.0193

Nguyen, K. T., Liang, W. K., Lee, V., Chang, W. S., Muggleton, N. G., Yeh, J. R., Huang, N. E., & Juan, C. H. (2019). Unraveling nonlinear electrophysiologic processes in the human visual system with full dimension spectral analysis. *Scientific Reports*, *9*(1). https://doi.org/10.1038/s41598-019-53286-z

Quinn, A., Lopes-dos-Santos, V., Dupret, D., Nobre, A., & Woolrich, M. (2021). EMD: Empirical Mode Decomposition and Hilbert-Huang Spectral Analyses in Python. *Journal of Open Source Software*, *6*(59), 2977. https://doi.org/10.21105/joss.02977
